# Analysis of Spatiotemporal Risks of Environmental Driving Factors of Respiratory Diseases

**DOI:** 10.1029/2026GH001881

**Published:** 2026-08-25

**Authors:** Xu Yang, Junshu Wang, Guoming Zhang, Zhaoyuan Yu

**Affiliations:** ^1^ State Key Laboratory of Climate System Prediction and Risk Management Nanjing Normal University Nanjing China; ^2^ Key Laboratory of Virtual Geographic Environment Nanjing Normal University Nanjing China; ^3^ Jiangsu Center for Collaborative Innovation in Geographical Information Resource Development and Application Nanjing China; ^4^ Health Information Center of Jiangsu Province Nanjing China

## Abstract

Respiratory diseases pose a serious threat to the health of residents in China and cause enormous socio‐economic losses. Jiangsu has a total of over 27 million vulnerable people in two groups: children under 15 years old and the elderly over 65 years old. Therefore, studies on the spatio‐temporal risks of respiratory diseases and their influencing factors in Jiangsu Province are of particular importance. Based on the Distributed Lag Nonlinear Model (DLNM) and the Geographically and Temporally Weighted Regression Model (GTWR), this study took the hospitalized cases of respiratory diseases in Jiangsu Province from 2020 to 2023 as research samples. It deeply explored the complex temporal relationships and spatial differences between environmental driving factors and the HRRD (hospitalization rate of respiratory diseases). It quantified the nonlinear relationships and lag effects of environmental driving factors on the HRRD. Additionally, it conducted a quantitative analysis of the specific impacts of each factor on the hospitalization rate under different spatio‐temporal conditions. The research results show that there are significant differences in the lag effects of extreme meteorological factors, air pollutants, and comprehensive meteorological factors on respiratory diseases among different age groups. Similarly, the impacts of these factors on the HRRD also exhibit different spatial heterogeneities. This research reveals the spatio‐temporal distribution patterns and driving mechanisms of respiratory diseases, offering a scientific foundation for the development of regionalized and differentiated prevention and control strategies.

## Plain Language Summary

Respiratory diseases pose a significant threat to public health and incur substantial socioeconomic costs, making it crucial to understand their spatio‐temporal risks and influencing factors, particularly in populous areas like Jiangsu Province, which is home to over 27 million vulnerable individuals including children under 15 and adults over 65.Using the Distributed Lag Nonlinear Model (DLNM) and Geographically and Temporally Weighted Regression (GTWR) on hospitalization data from 2020 to 2023, this study reveals that extreme meteorological factors, air pollutants, and composite indices like feels‐like temperature exhibit distinct age‐specific lag effects and spatial heterogeneities in their impact on respiratory disease hospitalization rates. For instance, older adults face peak risks from ${\text{highPM}}_{2.5}$ exposure 10–15 days later, while children show accumulating ozone‐related risks after 15 days; southern Jiangsu sees colder temperatures linked to higher hospitalizations, whereas northern regions show weaker temperature effects. These findings underscore the need for regionally tailored prevention strategies.

## Key Points

- Explored the complex spatiotemporal relationships and spatial differences between environmental factors and respiratory diseases
- Quantified nonlinear and lagged effects of environmental factors on respiratory diseases via distributed lag nonlinear model
- Quantified individual factors' specific impacts on regional respiratory disease via geographically and temporally weighted regression

## 1 Introduction

Against the backdrop of global climate change, the acceleration of urbanization, and the rapid development of social economy, severe weather and air pollution incidents have become more frequent (Alahmad et al., 2023; Cao et al., 2023; Yang et al., 2023). Extreme weather events have significantly affected the transmission and spread of infectious diseases, either directly or indirectly, through various mechanisms, presenting a grave hazard to public health (Anyamba et al., 2019; Chala & Hamde, 2021). Numerous studies have proven that adverse air and meteorological conditions not only lead to environmental disasters but also increase the risk of morbidity and mortality from chronic non‐communicable diseases and accelerate the spread of infectious diseases (T. Huang et al., 2024; Khomenko et al., 2023; Sharma et al., 2018). Respiratory diseases are one of the important causes of the global disease burden. In China, respiratory system diseases rank fourth among the causes of death of residents, accounting for 10.6% of the total deaths nationwide (Long et al., 2022). The treatment costs of respiratory system diseases are at a relatively high level and account for a large proportion of the total disease treatment costs, imposing a huge economic burden on society and the patients (Chai et al., 2015; Man et al., 2018; Tianli et al., 2020; Yang et al., 2017). Therefore, it is extremely urgent to study the risk factors affecting the incidence of respiratory diseases.

In recent years, a variety of statistical and spatio‐temporal analysis methods have been widely applied to study the influence mechanisms of driving factors of various diseases. These methods have made remarkable progress in revealing the linear and nonlinear relationships, lag effects, and spatial heterogeneity between respiratory system diseases and influencing factors. For example, X. Li et al. used the DLNM and meta‐analysis to explore the relationship between environmental temperature and emergency room visits for respiratory tract diseases in Beijing. Their research results showed that the cumulative lag effects of heat and cold effects peaked at a lag of 0–21 days, both of which would increase the risk of morbidity of respiratory system diseases(Li et al., 2024).Yi et al. established a GTWR model between the incidence of hand‐foot‐and‐mouth disease and climate factors, and found that there was a spatio‐temporal correlation between them, and the incidence of hand‐foot‐and‐mouth disease had regional differences and seasonal variation trends (Yi et al., 2021). Most of the existing studies explore the overall correlation between respiratory diseases in a single city or region and the influencing factors from the perspective of time series. They pay less attention to the differences in the effects of various factors across different regions, and there is also a lack of in‐depth analysis of the impact of urban socio‐economic factors.

Therefore, this study aims to investigate the complex temporal relationships and spatial differences between environmental driving factors and the HRRD. It seeks to quantify the nonlinear relationships and lag effects of environmental driving factors on the HRRD, and conduct a quantitative analysis of the specific impacts of various factors on the HRRD under different spatio‐temporal effects. In addition, a combined prediction model will be constructed to enable the predictive analysis of the hospitalization situation of respiratory diseases in Jiangsu Province. The research findings can provide a scientific basis for formulating targeted prevention and control strategies for respiratory diseases in Jiangsu Province and similar regions. By implementing differentiated health intervention measures for different regions and populations, it is expected to reduce the incidence and HRRD.

## 2 Materials and Method

### 2.1 Study Area

**Figure 1:**
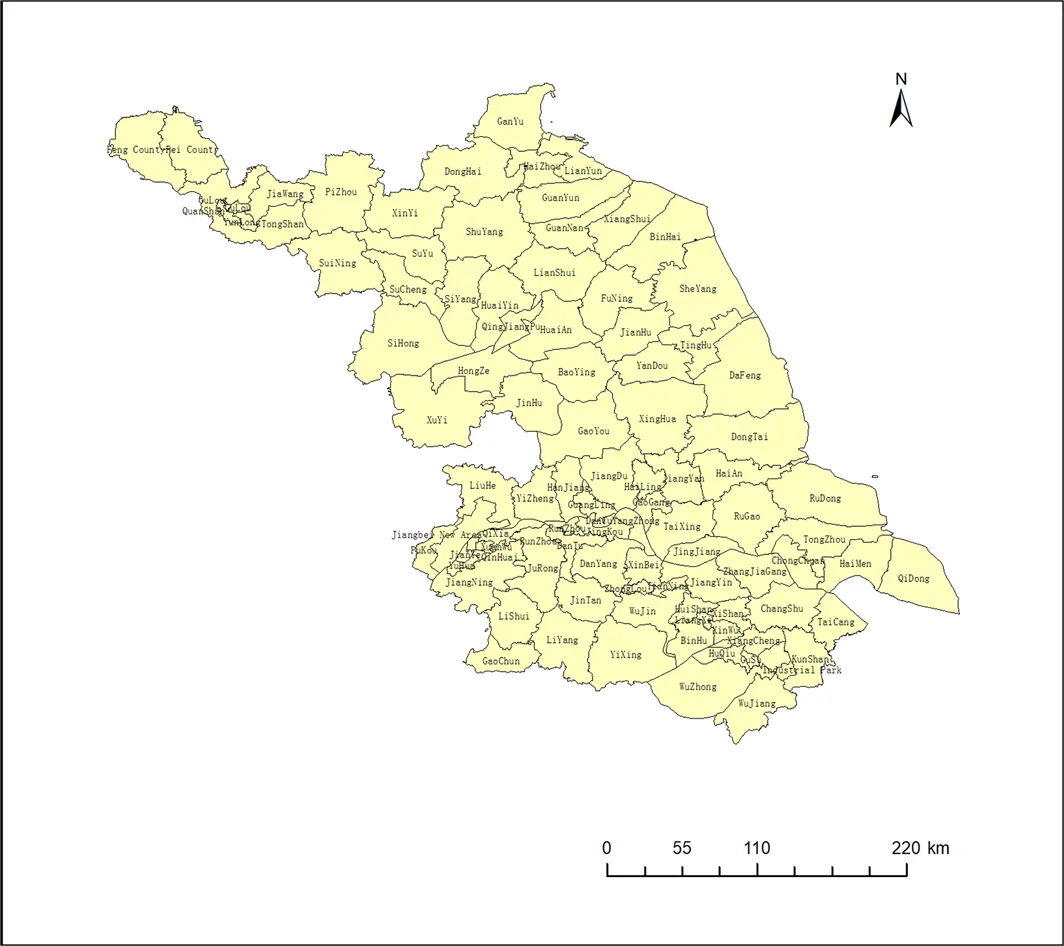
Administrative division map of counties and districts in jiangsu province.

The research area selected for this study is Jiangsu Province, China (Figure 1). This province is located in the core area of the Yangtze River Delta, with geographical coordinates ranging from 116${}^{\circ}\;18^{\prime }$ −121${}^{\circ}\;57^{\prime }$ E and 30${}^{\circ}\;45^{\prime }$ −35${}^{\circ}\;20^{\prime }$ N. The total area of Jiangsu Province is 107,200 square kilometers, presenting typical geographical characteristics of the north‐south transition zone. The unique geographical location of Jiangsu Province has given rise to its complex and diverse climate types. Taking the Huai River‐Subei Irrigation Main Canal as the boundary, the northern part belongs to a typical warm temperate semi‐humid monsoon climate, with distinct four seasons and cold and dry winters. The southern part, on the other hand, exhibits the characteristics of a subtropical humid monsoon climate, with simultaneous rainfall and heat and mild and humid winters (Qin et al., 2022; Sun et al., 2008). Such significant changes in the climatic gradient provide a natural experimental field for studying the relationship between meteorological factors and respiratory diseases. By the end of 2023, Jiangsu Province has 13 prefecture‐level cities and 95 counties (cities, districts) under its jurisdiction. The permanent resident population reaches as high as 85.26 million, among which the population aged 65 and above accounts for more than 18%, and the population of children aged 0–14 accounts for approximately 13% (J. P. B. of Statistics, 2024). The total number of these two groups of people who are susceptible to respiratory diseases exceeds 27 million, forming a large base of high‐risk populations.

### 2.2 Data Collection

This study incorporated six categories of data: demographic statistics, socioeconomic data, respiratory disease data, meteorological data, air pollutant data, and administrative boundary data. The data on respiratory diseases are the statistical data of inpatients with respiratory diseases from some hospitals in 89 counties and districts of Jiangsu Province (including Nanjing Jiangbei New Area and Suzhou Industrial Park; excluding Siyang County, Gaogang District, Yuhuatai District, Dantu District, Hongze District, Jinhu County, Xiangshui County and Wujin District) from 2020 to 2023. This data classifies and diagnoses respiratory diseases using ICD‐10 diagnostic codes (J00‐J06: acute upper respiratory tract infections; J09‐J18: influenza and pneumonia; J20‐J22: other acute lower respiratory tract infections), including information such as cities and counties, districts, dates, and the number of reported cases. By removing some abnormal data and data without regional attribution, we finally selected the daily records of 1,382,011 inpatients with respiratory diseases from 1 January 2020 to 31 December 2023 as the research data set. Meteorological data for the same period were sourced from the ERA5 data set (the fifth‐generation global atmospheric reanalysis product by the European Center for Medium‐Range Weather Forecasts, ECMWF) (Hersbach et al., 2023). The data set included parameters such as mean temperature, dew point temperature, and other relevant meteorological variables. The original meteorological data were provided in a 0.25° × 0.25° grid format. Relative humidity was scientifically derived from temperature and dew point temperature. Air pollutant data were obtained from the China High‐Resolution High‐Quality Near‐Surface Air Pollutants Data set, released by the National Tibetan Plateau Data Center. The data set included five major pollutants: ${\text{PM}}_{2.5}$, ${\text{PM}}_{10}$, $O_{3}$, ${\mathrm{SO}}_{2}$, and CO, with an original resolution of 1 km (Wei Jing, 2024). Socioeconomic data were collected from city‐ and county‐level statistical yearbooks, statistical bulletins on national economic and social development, and the Jiangsu Health Yearbook. The data covered total resident population, population density, per capita GDP, and the number of licensed physicians per 10,000 people. Administrative boundary data were sourced from the National Platform for Common Geospatial Information Services (with the boundaries of Nanjing Jiangbei New Area and Suzhou Industrial Park manually digitized by the authors).

### 2.3 Research Methods

Aiming at the characteristics that the impact of environmental driving factors on the respiratory diseases of the population has a certain degree of lag and spatial heterogeneity, this study, based on the DLNM and the GTWR, explores the complex temporal relationships and spatial differences between environmental driving factors and the HRRD. It quantifies the nonlinear relationships and lag effects of environmental driving factors on the HRRD, and conducts a quantitative analysis of the specific impacts of various factors on the HRRD under different spatio‐temporal effects. The DLNM, by integrating the lag effect and nonlinear relationship, quantifies the dynamic impacts of various environmental influencing factors on diseases. The GTWR further expands on the basis of geographically weighted regression, taking into account the dual heterogeneity of time and space simultaneously. The comprehensive application of these methods not only provides a multi‐dimensional perspective for revealing the driving mechanisms of respiratory diseases, but also lays a scientific foundation for formulating precise and regional prevention and control strategies.

#### 2.3.1 DLNM

The DLNM is a powerful statistical model used for analyzing the exposure‐response relationship. It is mainly applied to deal with the lagged and nonlinear impacts of exposure factors (such as air pollution, climate change, etc.) on health outcomes or other response variables (such as disease incidence rates, changes in economic indicators, etc.) (Hu et al., 2018; Yunquan et al., 2017). According to existing studies, meteorological factors have a nonlinear impact on respiratory diseases and exhibit a lag effect. In addition, in order to address the problem of overdispersion, we adopt the quasi‐Poisson model as the link function of the fitting model. At the same time, the influences of factors such as long‐term trends, weekly effects, and holidays are taken into consideration. The basic expression of the DLNM (Gasparrini et al., 2010) is as follows:

$$\log\left[E\left(Y_{i}\right)\right]=\alpha +cb\left(X_{i}\right)+ns\left(Time,df\right)+{Dow}_{i}+{Holiday}_{i}$$

Among them, t represents the observation date, $Y_{t}$ represents the number of hospitalized cases of respiratory diseases on the t‐th day, α represents the intercept; the cross‐basis matrix cb represents the relationship between respiratory diseases and various meteorological factors (such as temperature, air pressure, and ${\text{PM}}_{2.5}$). The variable $X_{i}$ represents these meteorological factors, and ns represents the natural cubic spline function used to capture complex relationships. The time components include seasonality and long‐term trends to check whether the experimental results are periodic. In addition, the variables ${\text{Dow}}_{t}$ and ${\text{Holiday}}_{t}$ represent the day of the week and public holidays respectively. In the variable ${\text{Holiday}}_{t}$, holidays are set to 1 and working days are set to 0. As for the variable ${\text{Dow}}_{t}$, Monday is represented as 1, Tuesday is represented as 2, and so on. The impacts of meteorological and pollutant factors on respiratory diseases are expressed as the relative risk (RR), which is calculated by the following formula:

$$RR=W^{p}\hat{\eta }$$

$$\mathrm{SE}=\sqrt{\mathrm{diag}\left(W^{p}V\left(\hat{\eta }\right)W^{pT}\right)}$$

$$95\%CI=\left(e^{\left(RR-1.96\times \mathrm{SE}\right)},e^{\left(RR+1.96\times \mathrm{SE}\right)}\right)$$

$\hat{\eta }$ is a vector of estimated coefficients obtained from the fitted model, which includes the cross‐basis function matrix W; $W^{p}$ is the cross‐basis function matrix at lag p. SE is the standard error of RR; $V\left(\hat{\eta }\right)$ is the variance‐covariance matrix of the estimated coefficients $\left(\hat{\eta }\right)$; $W^{pT}$ is the transpose of the cross‐basis function matrix at lag p; $\sqrt{\mathrm{diag}\left(\text{…}\right)}$ represents taking the square root of the diagonal elements of the matrix.

The degrees of freedom (df) for each variable are determined by fitting the Q‐AIC. The degrees of freedom for time are set to 7. In addition, taking into account the characteristics of respiratory system diseases and the insights from previous studies, a 30‐day lag with a relatively low Q‐AIC value is set to explore the potential lag relationships.

#### 2.3.2 GTWR

Spatio‐temporal geographically weighted regression is an extension of Geographically Weighted Regression (GWR) in the spatio‐temporal dimension. Different geographical locations. GTWR further considers the changes in th GWR assigns local weights based on the geographical locations for each observation point, enabling the model to take into account spatial non‐stationarity, that is, the relationships between variables may vary ate temporal dimension on this basis, assigning spatio‐temporal weights to each observation point at different time points(Fotheringham et al., 2015). It assumes that the relationships between variables not only differ spatially but also at different time points, and reflects these spatio‐temporal variation characteristics through a spatio‐temporal weight matrix, thus more precisely depicting the spatio‐temporal heterogeneity of the relationships between variables. The expression formula is as follows:

$$y_{i}=\beta_{0}\left(u_{i},v_{i},t_{i}\right)+\sum_{k=1}^{p}\beta_{k}\left(u_{i},v_{i},t_{i}\right)X_{ik}+\varepsilon_{i}$$

Among them, $y_{i}$ represents the incidence rate of the i‐th sample, p is the number of factors, $u_{i}$ and $v_{i}$ respectively represent the longitude and latitude coordinates of the i‐th sample, $t_{i}$ represents the time coordinate of the i‐th sample, $\left(u_{i},v_{i},t_{i}\right)$ represents the spatio‐temporal dimension coordinates of the i‐th sample point, $\beta_{0}\left(u_{i},v_{i},t_{i}\right)$ is the constant term, $\beta_{k}\left(u_{i},v_{i},t_{i}\right)$ represents the regression coefficient of the k‐th explanatory variable at the i‐th sample point, $X_{ik}$ represents the k‐th independent variable of the i‐th sample point, and $\varepsilon_{i}$ represents the random error. The estimation of $\beta_{k}\left(u_{i},v_{i},t_{i}\right)$ is expressed as follows:

$$\hat{\beta }\left(u_{i},v_{i},t_{i}\right)={\left[X^{T}W\left(u_{i},v_{i},t_{i}\right)X\right]}^{-1}X^{T}W\left(u_{i},v_{i},t_{i}\right)Y$$

In the formula, $W\left(u_{i},v_{i},t_{i}\right)=\mathrm{diag}\left(\alpha_{i1},\;\alpha_{i2},\;\text{…},\;\alpha_{in}\right)$, and n is the number of samples. $\alpha_{ij}\;\left(1\leq j\leq n\right)$ is a spatio‐temporal distance function of $\left(u_{i},v_{i},t_{i}\right)$, corresponding to the weights in the weighted regression adjacent to the sample point i. To improve the goodness of fit of the model, it is assumed that the observed data closer to the observation point i have a greater impact on the parameter estimation of $\beta_{k}\left(u_{i},v_{i},t_{i}\right)$. Here, “closer” means proximity not only in geographical distance but also in temporal distance.

## 3 Results and Discussion

### 3.1 Spatio—Temporal Analysis of Respiratory Diseases

**Figure 2:**
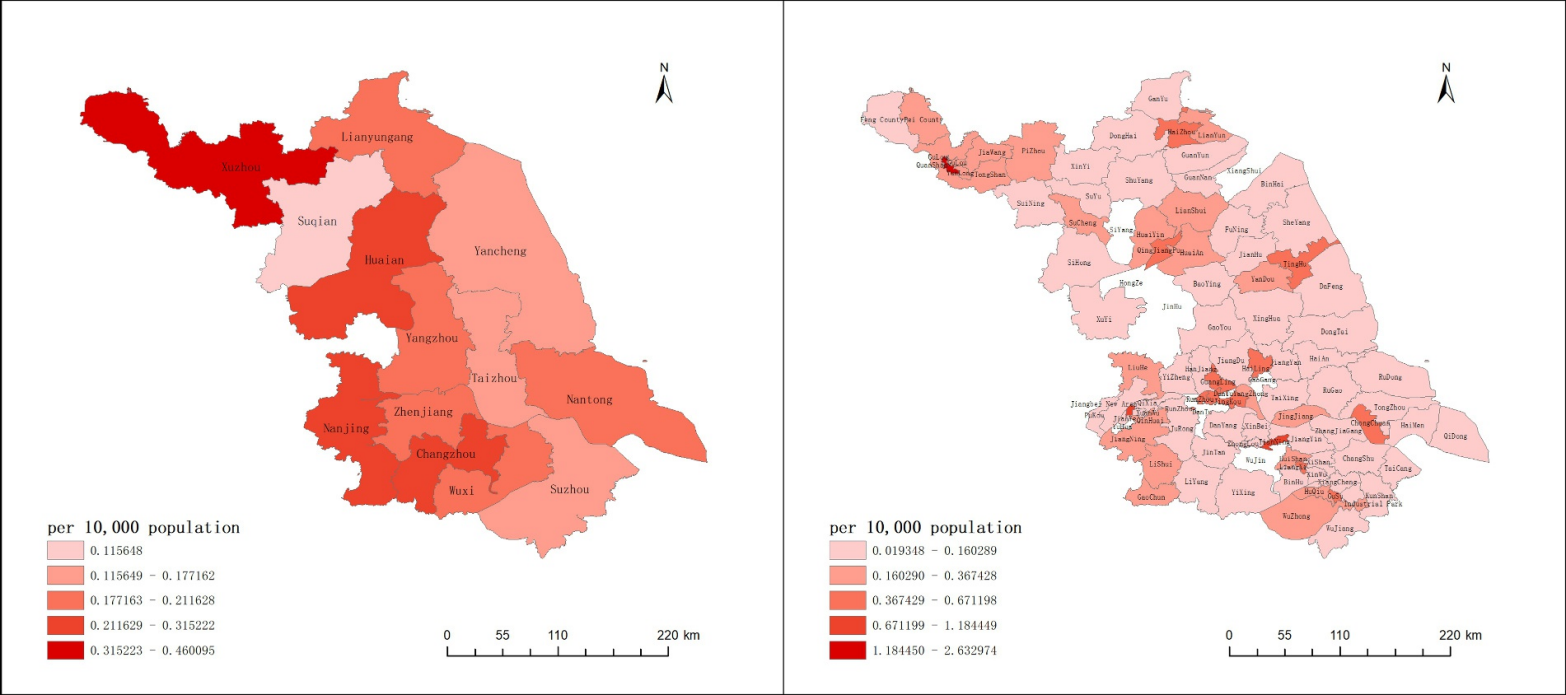
Spatial Distribution Map of Hospitalization Rates for Respiratory Diseases (Left: 13 Prefecture ‐ level Cities; Right: 89 Counties and Districts).

From 1 January 2020, to 31 December 2023, a total of 2,310,947 hospitalized cases of respiratory diseases were reported in 89 counties and districts of Jiangsu Province, with an average of 1,582 hospitalized cases per day. Among them, there were 1,315,201 hospitalized male cases and 995,686 hospitalized female cases, with a male‐to‐female ratio of 1.32:1. There were 1,268,368 cases of patients under 15 years old, accounting for 54.89%; 471,966 cases of patients aged 15 to 65, accounting for 20.42%; and 570,613 cases of patients over 65 years old, accounting for 24.69%. From the perspective of prefecture‐level cities, among the 13 cities in Jiangsu Province, Xuzhou City reported the largest number of cases, reaching 419,748, while Suqian City reported the smallest number of cases, which was 56,979. Figure 2 shows the spatial distribution of the daily average HRRD in 13 cities of Jiangsu Province from 2020 to 2023. It can be seen from the figure that Xuzhou City has the highest hospitalization rate, and Suqian City has the lowest hospitalization rate. As shown in the right part of Figure 2, the areas with a higher HRRD are often geographically adjacent, such as Quanshan, Gulou, and Jiawang Districts in northern Jiangsu, Guangling, Dantu, and Runzhou Districts in the central part, as well as Jianye, Qinhuai, Jiangbei New Area, and Tianning, Huishan, Wuzhong, and Industrial Park Districts in the southern part. This indicates that there are similar factors affecting the HRRD in these clustered areas, such as similar geographical environments, climatic conditions, and economic development situations, resulting in a certain similarity in the risk of residents suffering from respiratory diseases. In addition, factors such as population mobility between regions and the degree of sharing of medical resources may also have an impact, indicating that the incidence rate of respiratory diseases in one region can be affected by surrounding areas.

**Figure 3:**
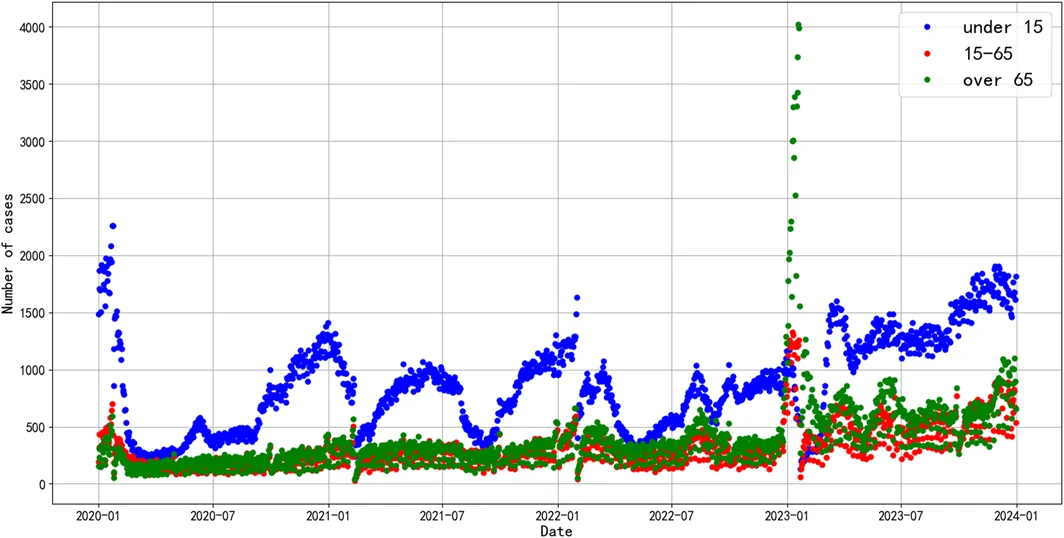
Time series diagram of the daily number of inpatient cases of respiratory diseases in jiangsu province.

Figure 3 is a time series diagram of the daily number of inpatient cases of respiratory diseases in Jiangsu Province. As can be seen from the figure, people of different age groups all exhibit certain periodic patterns, especially those under 15 years old. The number of cases of respiratory diseases in the three age groups all showed obvious peaks at the beginning of 2020. Subsequently, from the middle of 2020 to the end of 2022, the number of cases was relatively stable, showing certain seasonal fluctuation characteristics. The number of cases increased slightly every winter (around December to February of the following year). From the end of 2022 to the beginning of 2023, the number of cases among people over 65 years old increased significantly, reaching extremely high values. This may be due to the change of relevant prevention and control policies at the end of 2022, which led to an increase in the risk of virus transmission. In addition, the elderly population, as a susceptible group to respiratory diseases, has a relatively weak immune system and often has underlying diseases. Therefore, after the policy adjustment, they are more vulnerable to virus attacks, resulting in the phenomenon of concentrated illness and hospitalization. In addition, winter is originally a peak period for respiratory diseases. Factors such as low temperature, dry climate conditions, and insufficient indoor ventilation further increase the health risks of the elderly population.

**Figure 4:**
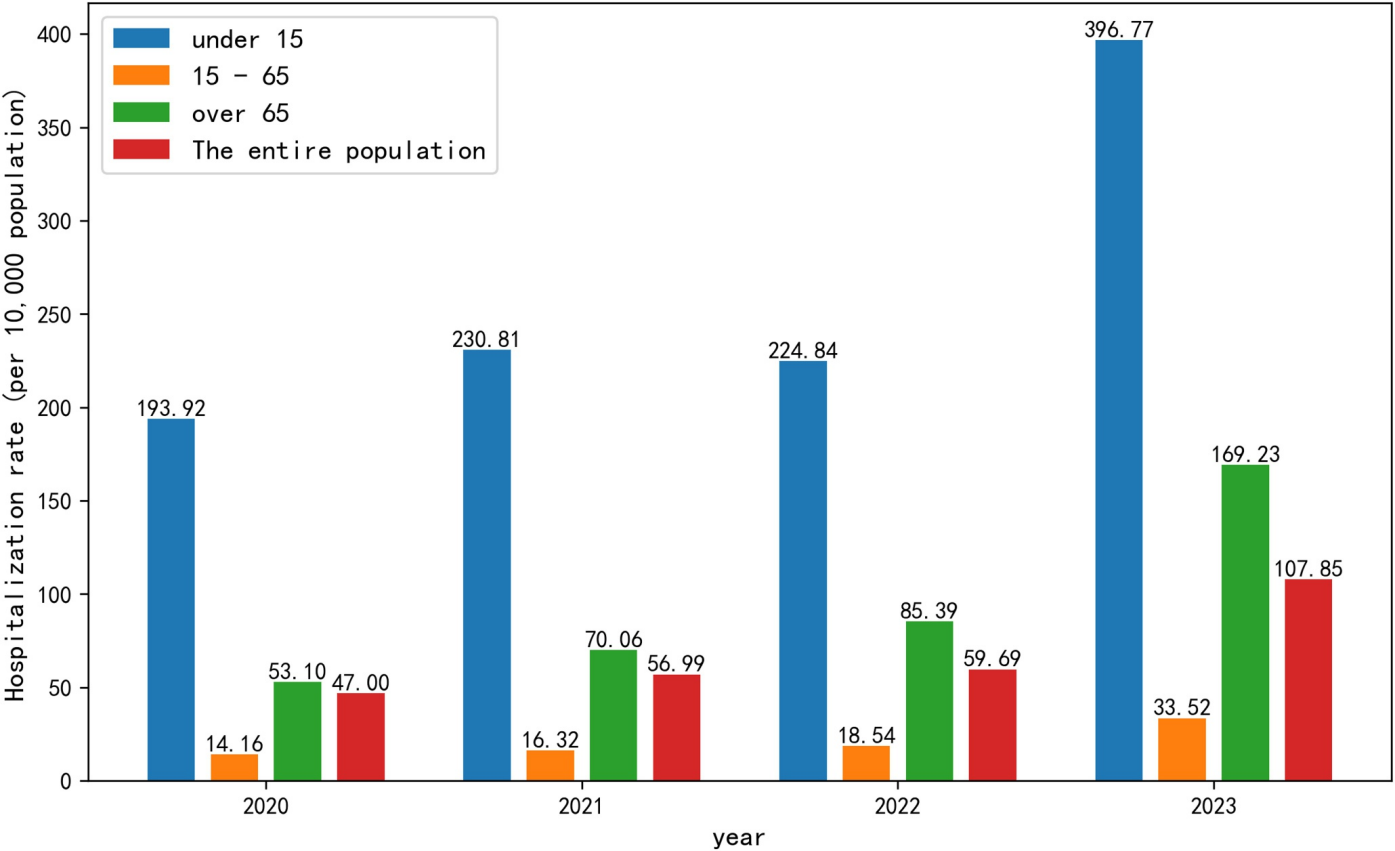
Time series diagram of the distribution of inpatient rates of respiratory diseases in different age groups in jiangsu province from 2020 to 2023.

**Figure 5:**
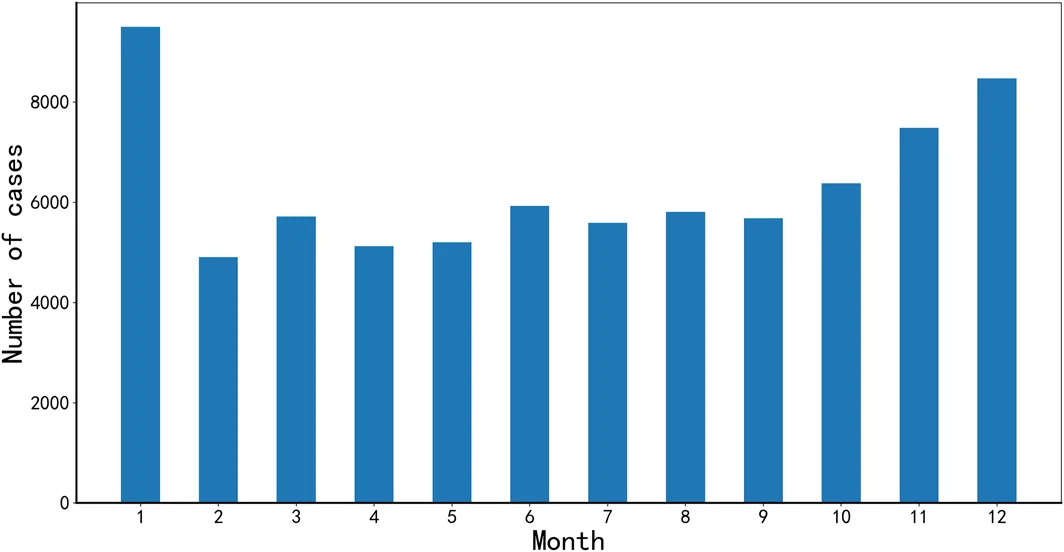
Average daily hospitalized cases of respiratory diseases by month in Jiangsu Province.

Figure 3 visually presents the dynamic changes in the daily number of inpatient cases of respiratory diseases in Jiangsu Province over time, clearly revealing the periodic and seasonal fluctuation characteristics of the number of cases across different age groups, as well as the abnormal growth trend after the end of 2022. To further quantify this change pattern and focus on age differences, Figure 4, with the year as the time unit, shows the changes in the hospitalization rate of different age groups in Jiangsu Province from 2020 to 2023, highlighting the ranking of hospitalization rates among various age groups and their annual changing trends. The data indicates that over these four years, the hospitalization rate of the population under 15 years old ranks the highest, while that of the population aged 15–65 years old is the lowest. From the perspective of time dimension, the hospitalization rate of the population under 15 years old only experiences a slight decline in 2022, and an upward trend year by year is shown in the hospitalization rates of other age groups as well as the entire population. Among them, the growth in 2021 and 2022 is relatively gentle, but in 2023, the hospitalization rates of all populations climb significantly, especially for the population over 65 years old, with a growth rate as high as 98.2% compared to the previous year. Figure 5 shows the monthly distribution of the daily average number of hospitalized cases of respiratory diseases. It can be clearly seen that the daily average number of hospitalized cases reaches its peak in January, followed by November and December, while it is relatively low in February, April, and May. The daily average number of hospitalized cases in the remaining months fluctuates slightly and remains relatively stable as a whole.

### 3.2 Spatio‐Temporal Analysis of Meteorological Factors and Air Pollutants

**Figure 6:**
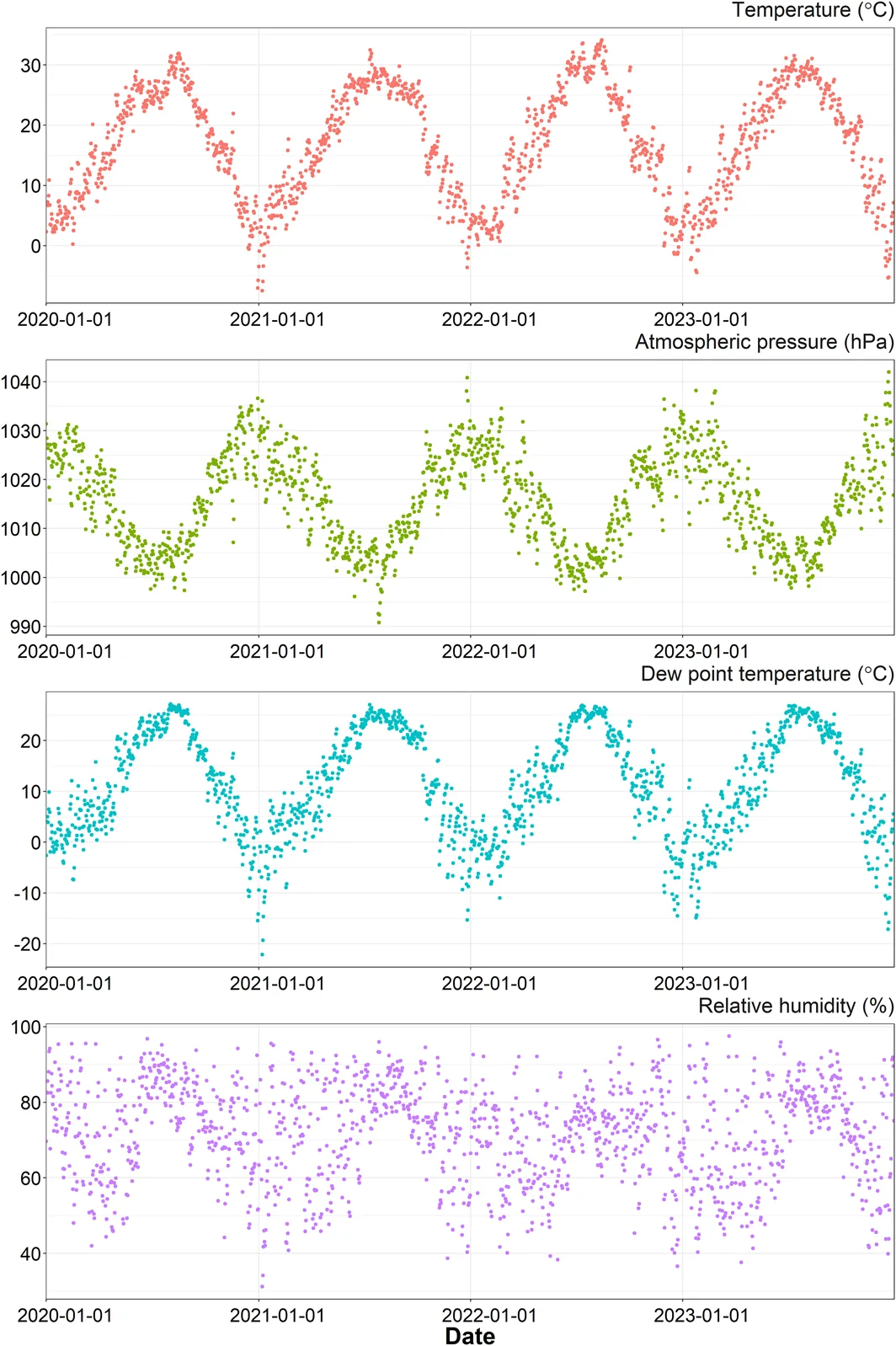
Time series diagram of meteorological factors in jiangsu province.

During the study period, the data indicators of meteorological factors and air pollutants in Jiangsu Province are shown in Table 1. As an economically developed region along the eastern coast of China, the air pollutants in Jiangsu Province mainly come from industrial emissions, transportation sources, energy consumption, agricultural activities, and natural sources. As shown in Figure 6, temperature, air pressure, and dew point temperature exhibit obvious seasonal periodic changes. Specifically, the interannual change trends of temperature and dew point temperature are highly consistent. Both reach their troughs in winter (from December to February of the following year) and peak in summer (from June to August), and this characteristic of synchronous change fully reflects the physical dependence between water vapor pressure and temperature. In sharp contrast is the change pattern of air pressure, and its seasonal fluctuation has a significant negative correlation with temperature: it is controlled by the cold high‐pressure system in winter and affected by the subtropical low‐pressure system in summer.

**Table 1:** Descriptive Statistics of Variables

Variable	Mean	Min	Median	Max	Std
Daily hospitalizations	18.49	0	10	569	28.32
Under 15 years old	10.15	0	5	376	18.75
15–65 years old	3.78	0	2	122	5.68
Over 65 years old	4.57	0	2	419	8.37
Temperature(°C)	16.76	−7.46	17.15	34.12	9.33
Pressure(hPa)	1015.43	990.75	1015.71	1041.97	9.65
RH(%)	71.80	31.17	73.41	97.52	13.03
DT(°C)	11.39	−22.16	11.47	27.17	10.33
CO(mg/$m^{3}$)	0.34	0.20	0.32	0.68	0.07
$O_{3}$(μg/$m^{3}$)	53.14	12.73	51.34	111.70	18.67
${PM}_{10}$(μg/$m^{3}$)	28.04	5.39	24.12	161.60	15.29
${PM}_{2.5}$(μg/$m^{3}$)	15.63	2.85	12.97	68.32	9.04
${SO}_{2}$(μg/$m^{3}$)	4.00	2.39	3.80	8.03	0.92

**Figure 7:**
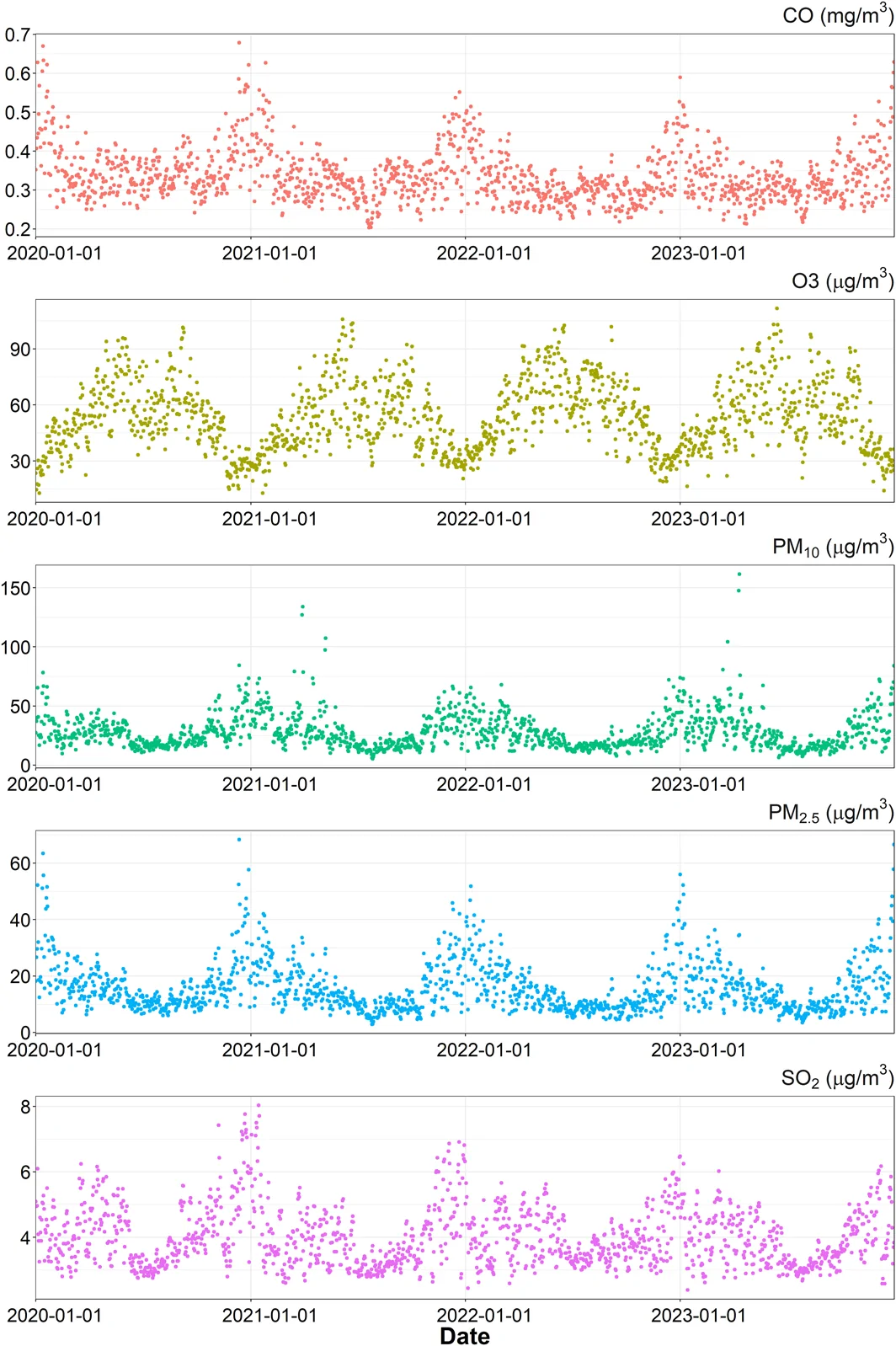
Time series diagram of atmospheric pollutants in jiangsu province.

As can be seen from Figure 7, five air pollutants all exhibit seasonal periodic changes to varying degrees. Among them, ozone ($O_{3}$) shows a typical distribution of “higher in summer and lower in winter”, and this distribution characteristic highly coincides with the seasonal changes of meteorological elements such as solar radiation intensity and temperature. Strong solar radiation not only directly promotes the rate of photochemical reactions, but high‐temperature conditions are also conducive to the volatilization of volatile organic compounds, providing sufficient precursors for ozone generation. The remaining four pollutants (CO, ${\text{PM}}_{10}$, ${\text{PM}}_{2.5}$, ${\text{SO}}_{2}$) all show an anti‐phase change of “higher in winter and lower in summer”. The seasonal change trend of ${\text{PM}}_{10}$ is relatively weak, and the concentration fluctuates less throughout the year, which may be greatly affected by non‐seasonal emission sources such as dust weather and construction dust. The seasonal fluctuations of ${\text{PM}}_{2.5}$ may be mainly due to the combined effects of multiple factors such as increased emissions from coal‐fired heating in winter, a decrease in the height of the atmospheric boundary layer, and an increase in the frequency of temperature inversion. The high values of ${\text{SO}}_{2}$ in winter may be related to the increase in fossil fuel combustion and adverse meteorological conditions, while the seasonal changes of CO reflect the characteristic of a large number of motor vehicles in Jiangsu Province. The low temperature in winter leads to a decrease in combustion efficiency and an increase in exhaust emissions. These findings together reveal the complexity and seasonal characteristics of the formation mechanism of air pollution in Jiangsu Province.

**Figure 8:**
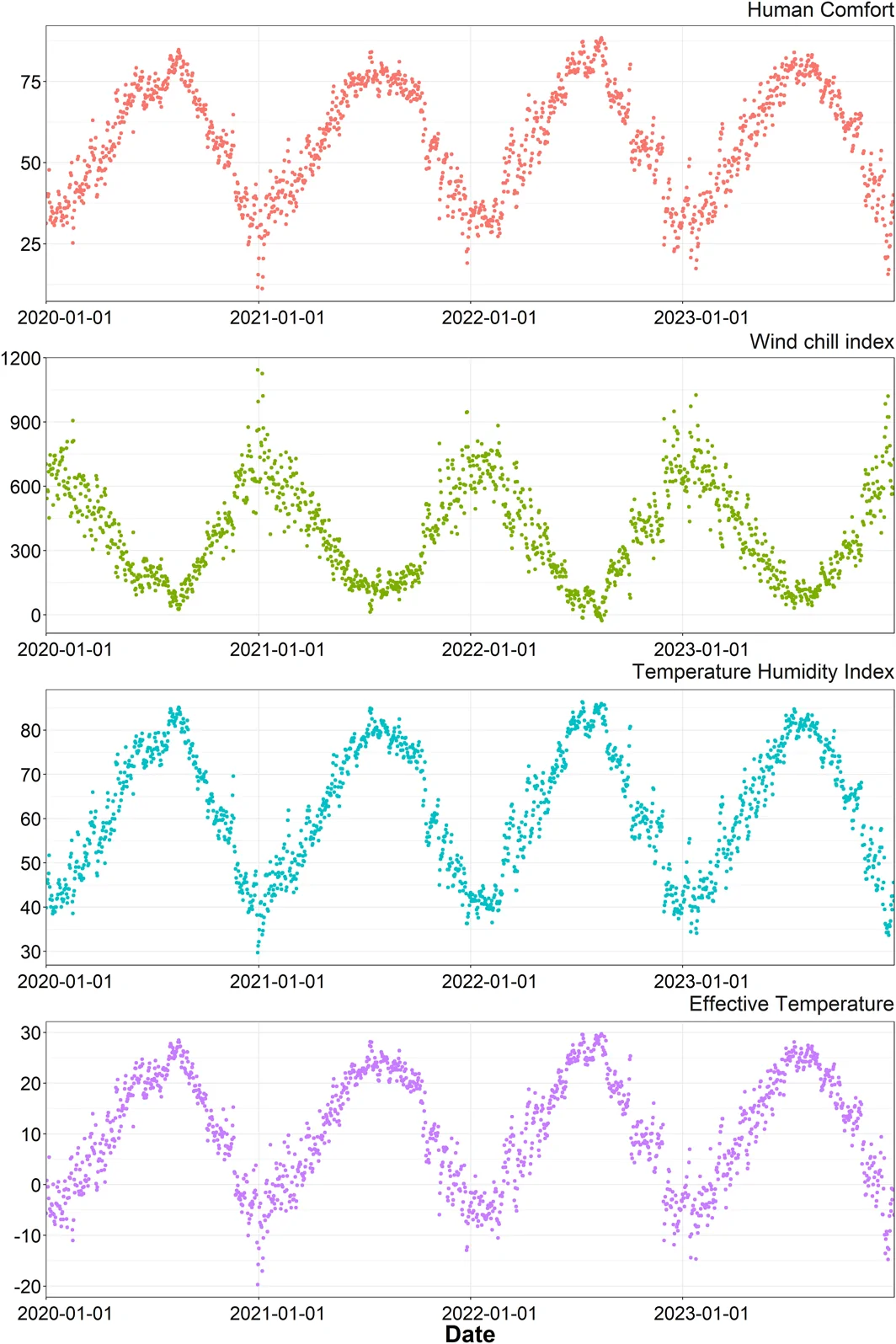
Time series diagram of comprehensive meteorological indicators in jiangsu province.

It can be clearly observed from Figure 8 that the four comprehensive meteorological indicators all exhibit significant seasonal variation patterns. Specifically, the variation trends of human comfort, the temperature‐humidity index, and the perceived temperature are relatively consistent, all showing the characteristics of being lower in winter and higher in summer. This variation is closely related to the seasonal fluctuations of temperature: the high temperature and high humidity environment in summer significantly increases human comfort and the temperature‐humidity index, while the low temperature and low humidity conditions in winter lead to a significant decrease in these indicators. In contrast, the variation trend of the wind chill index is opposite to the above three indicators, showing the characteristics of being higher in winter and lower in summer. This is mainly because the combined effect of low temperature and wind speed in winter exacerbates the human feeling of cold, while the influence of the wind chill index is relatively weak in the high temperature environment in summer. The seasonal changes of these comprehensive meteorological indicators not only reflect the natural laws of climate but are also closely related to the changes in human activities and the ecological environment. For example, the increase in human comfort and the temperature‐humidity index in summer may be related to the heat island effect during the urbanization process, and the significant increase in the wind chill index in winter may be affected by the frequent occurrence of extreme weather events.

**Figure 9:**
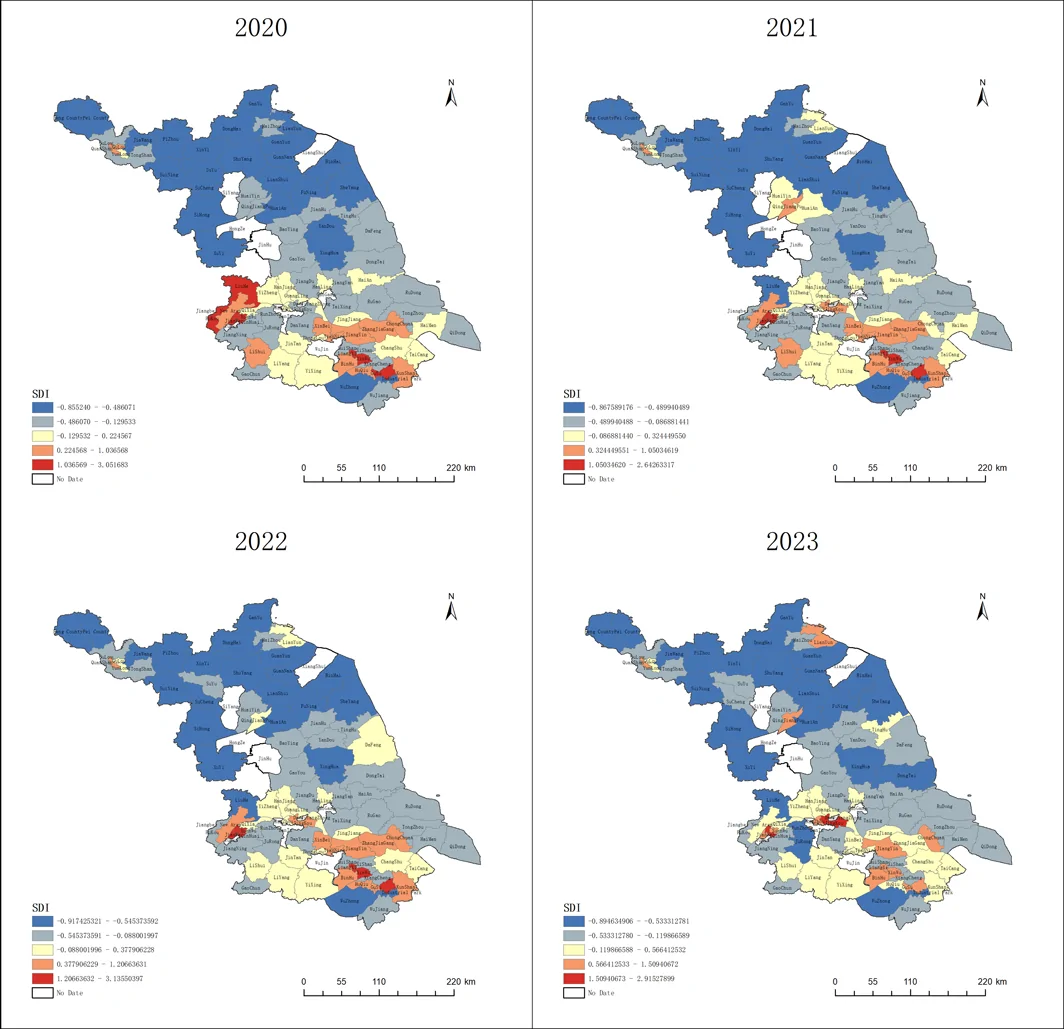
Spatial distribution map of SDI in jiangsu province.

Four indicators were selected for the calculation of the Social Development Index (SDI), including the permanent resident population, per capita GDP, population density, and the number of practicing physicians per capita. According to the results of the principal component analysis, three principal components were selected to calculate the SDI, which cumulatively explain 93.8% of the original index data, indicating that the SDI can, to a certain extent, represent the social development status of each county and district (Li et al., 2009). From 2020 to 2023, the SDI results of the 89 counties and districts in Jiangsu Province included in the study are shown in Figure 9. Overall, the SDI in southern Jiangsu is higher than that in northern Jiangsu, indicating that the urban development status in the northern region is generally lower than that in the southern region, which is highly consistent with the regional economic pattern of “strong in the south and weak in the north” in Jiangsu Province (Fotheringham et al., 2015). Among them, the SDI values of Gulou District, Qinhuai District, and Liangxi District are relatively high and have ranked among the top five over the 4 years, indicating that these areas have obvious advantages in economic development, medical resources, population agglomeration, and other aspects. The SDI values of Donghai County, Suining District, and Feng County are relatively low and have been among the last five over the 4 years, reflecting the comprehensive shortcomings of these areas in multiple dimensions of social development.

### 3.3 Analysis of the Time—Lag Effect of Influencing Factors of Respiratory Diseases

According to relevant epidemiological studies, the incidence of respiratory diseases is significantly correlated with extreme meteorological conditions (He et al., 2023). In order to deeply explore the impact degree of extreme meteorological factors on respiratory diseases, this study adopts the percentile method, selects the 5th percentile (P5) and the 95th percentile (P95) of each influencing factor as the definition thresholds of extreme meteorological conditions, and takes the median (P50) as the reference benchmark. By calculating the relative risk (RR), this study quantifies and evaluates the risks of respiratory diseases among people of different age groups under extreme meteorological conditions, and comprehensively aggregates and conducts heterogeneity tests on the results of each county and district through Meta‐analysis, so as to systematically reveal the impact mechanism and the effect intensity of extreme meteorological conditions in Jiangsu Province on respiratory diseases.

**Figure 10:**
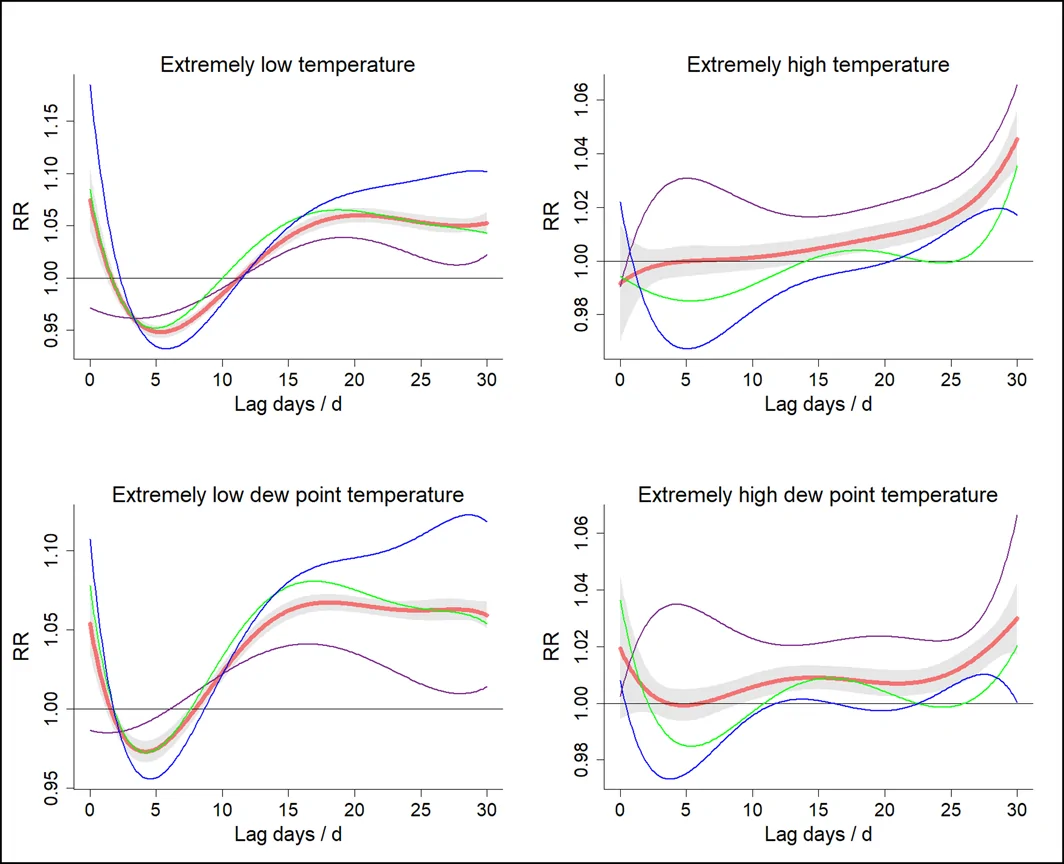
Analysis of the distributed lag effect of extreme air temperatures and dew point temperatures on respiratory diseases at different lag days (Red: The entire population; Purple: Population under 15 years old; Green line: Population aged 15–65 years old; Blue: Population over 65 years old).

As can be seen from Figure 10, except for the population under 15 years old, the relative risk change trends of respiratory diseases among people of other age groups under the influence of extremely low temperatures are similar, and their curves are all in a “U” shape. When the lag days are short, the relative risk is high, then it decreases, reaching a trough at about 5–10 days, and then gradually increases. For the population under 15 years old, the relative risk slowly increases with the lag days, reaches a peak at 15–20 days, and then gradually decreases. The reasons may be as follows: the immune system of children is in the developmental stage, and it takes a long process for the initiation and exertion of the immune response in the face of cold stimuli (Wu et al., 2024). Due to the high attention paid by parents and schools to children, effective warming measures are generally taken in winter, such as adding clothes and using heating equipment. These protective measures can delay the occurrence of the risk of respiratory diseases caused by low temperature exposure to a certain extent. At the same time, children's outdoor activity time in the cold season is significantly reduced, and their main activity places are concentrated in the indoor environment, and this spatial limitation also reduces the risk of direct exposure to extremely low temperatures (Qi et al., 2024). For the population over 65 years old, the relative risk is the highest (RR = 1.20) in the short lag days under the influence of extremely low temperatures, and the relative risk fluctuates significantly throughout the process of lag days change. This indicates that the population of this age group is highly sensitive to extremely low temperatures, and the impact on them is extremely complex. This may be because with the increase of age, the body's thermoregulatory function gradually declines, and the immune response ability decreases, leading to a significant reduction in the ability to adapt to the cold environment (Seltenrich, 2015). In addition, the coexistence of multiple diseases commonly existing in the elderly population further exacerbates the health risks caused by low temperatures. Specifically, low temperature exposure can cause peripheral vasoconstriction, lead to significant fluctuations in blood pressure, increase the afterload of the heart, and thus induce cardiovascular events; at the same time, the cold environment will reduce the defensive function of the respiratory mucosa, increase the susceptibility to pathogens, and lead to an increased risk of respiratory tract infections (Gasparrini et al., 2015). It is worth noting that the interaction between underlying diseases and the low temperature environment may produce a synergistic effect, which can explain the complexity of the relative risk fluctuations in the elderly population to a certain extent (Dominici et al., 2006). The population most at risk under the influence of extremely high temperatures is the population under 15 years old. The relative risk reaches a small peak (RR = 1.03) at about 5 lag days, and then fluctuates down and then up. For the population of other age groups, the relative risk is high at 0–2 lag days, then decreases rapidly, reaches the lowest point at 4–5 days, and then slowly increases and tends to be stable. This difference may be due to the age‐specific response mechanism of the respiratory system to high temperature stress. Since the respiratory tract structure of children is not fully developed, the airway is relatively narrow, and the mucosal barrier function is weak, phenomena such as dehydration of the respiratory tract and impaired mucociliary clearance are more likely to occur in a high‐temperature environment. The occurrence of the first risk peak may be related to the accumulation of respiratory tract inflammatory responses caused by heat stress, and the subsequent risk fluctuations reflect the dynamic response process of the children's immune system to respiratory tract infections (Sheffield & Landrigan, 2010). The influence of the dew point temperature and the air temperature on the hospitalization risk of respiratory diseases presents similar time‐risk distribution characteristics.

**Figure 11:**
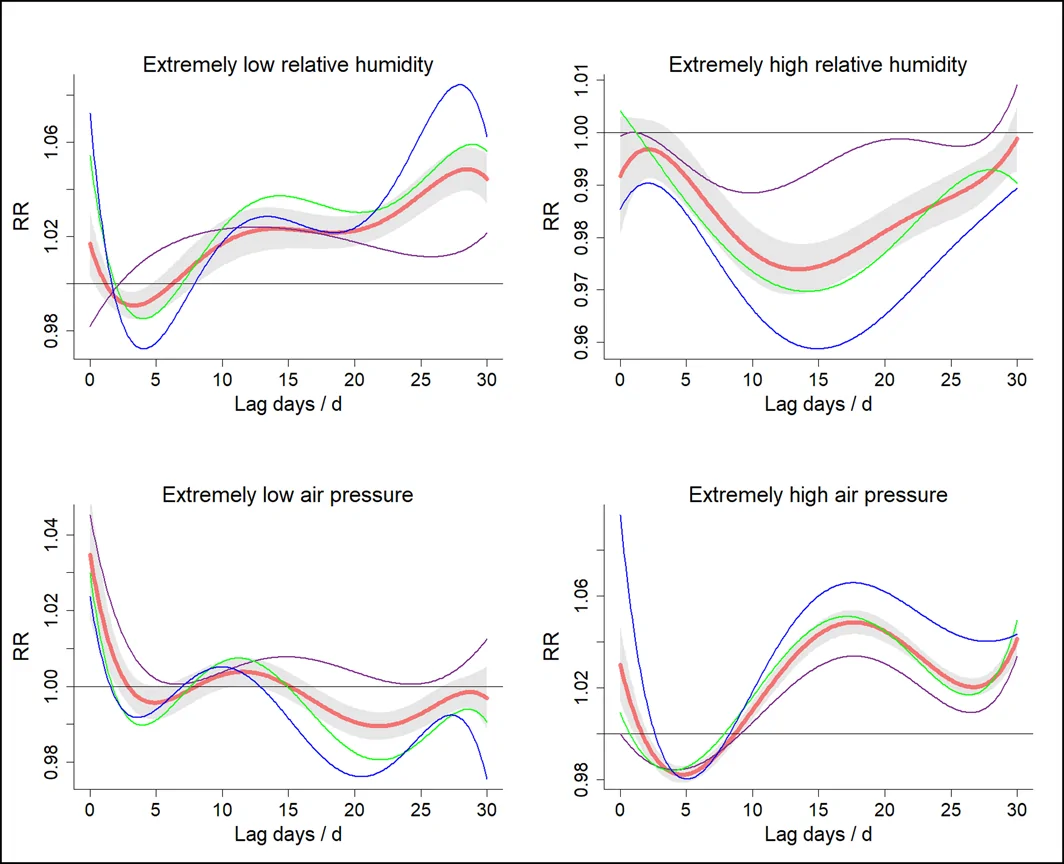
The distributed lag effect of extreme humidity and air pressure on respiratory diseases at different lag days (Red: The whole population; Purple: The population under 15 years old; Green line: The population aged 15–65 years old; Blue: The population over 65 years old).

Figure 11 shows that the responses of people of different age groups to the risk of respiratory diseases caused by extremely low humidity present different changes. The relative risk of respiratory diseases of the population under 15 years old affected by extremely low humidity gradually increases with the exposure time and tends to be stable after about 10 days. For the remaining age groups, the relative risk is relatively large on the same day, then decreases rapidly, and starts to increase continuously after 4–5 days. Under the influence of extremely high humidity, within 30 lag days, the relative risk values of people of all age groups are generally lower than 1, which means that the environment with extremely high humidity may have a protective effect on respiratory diseases. This may be related to multiple mechanisms such as maintaining the integrity of the respiratory mucosa, enhancing local immune function, and inhibiting the spread of pathogens (Guarnieri et al., 2023). The relative risk value of respiratory diseases of the population under 15 years old affected by extremely low air pressure is relatively stable. For the remaining age groups, the risk reaches its maximum on the same day and then fluctuates and decreases over time. The patterns of the temporal changes in the health impacts of extremely high air pressure on people of different age groups are similar, all showing the characteristics of nonlinear fluctuations, that is, first decreasing, then increasing, and then decreasing. Among them, the elderly population over 65 years old is the most sensitive to extremely high air pressure, and the hospitalization risk of respiratory diseases shows a significant upward trend. This may be due to the decline in lung function and the increased airway sensitivity of the elderly, making it difficult for them to adapt to the respiratory load brought about by the change in air pressure (Fusco et al., 2001).

**Figure 12:**
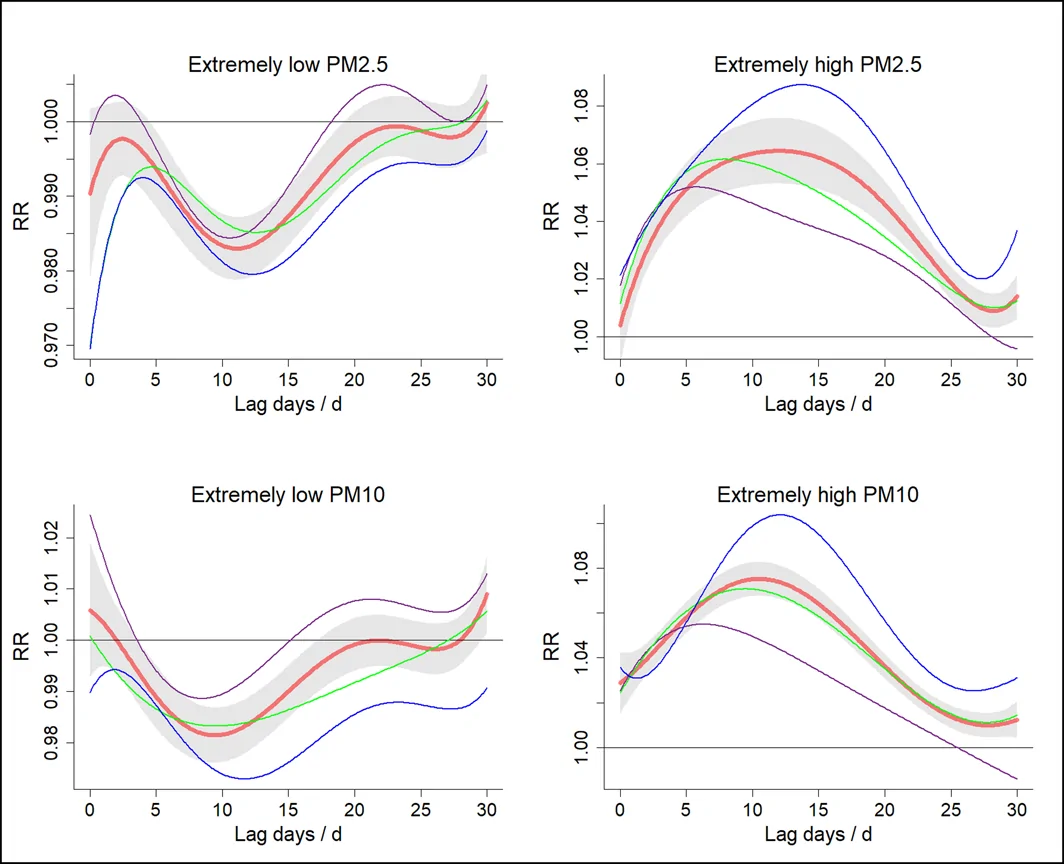
The distributed lag effect of extreme ${\text{PM}}_{2.5}$ and ${\text{PM}}_{10}$ on respiratory diseases at different lag days (Red: The entire population; Purple: The population under 15 years old; Green line: The population aged 15–65 years old; Blue: The population over 65 years old).

As can be seen from Figure 12, extremely low concentrations of ${\text{PM}}_{2.5}$ and ${\text{PM}}_{10}$ generally have a protective effect on the hospitalization risk of respiratory diseases within the range of 0–30 lag days. Exposure to high concentrations of ${\text{PM}}_{2.5}$ and ${\text{PM}}_{10}$ can significantly increase the hospitalization risk of respiratory diseases, and the elderly population over 65 years old is particularly sensitive. This impact reaches its peak 10–15 days after exposure. It is worth noting that there are differences in the action patterns of ${\text{PM}}_{10}$ and ${\text{PM}}_{2.5}$: The acute effect of ${\text{PM}}_{10}$ is more obvious, and an increase in the hospitalization risk can be observed on the day of exposure. This may be because particulate matter with larger particle sizes in ${\text{PM}}_{10}$ is more likely to deposit in the upper respiratory tract, triggering acute inflammatory reactions. In contrast, since ${\text{PM}}_{2.5}$ can penetrate deep into the alveoli, its pathogenic effects often require a longer incubation period to manifest (Wu et al., 2018).

**Figure 13:**
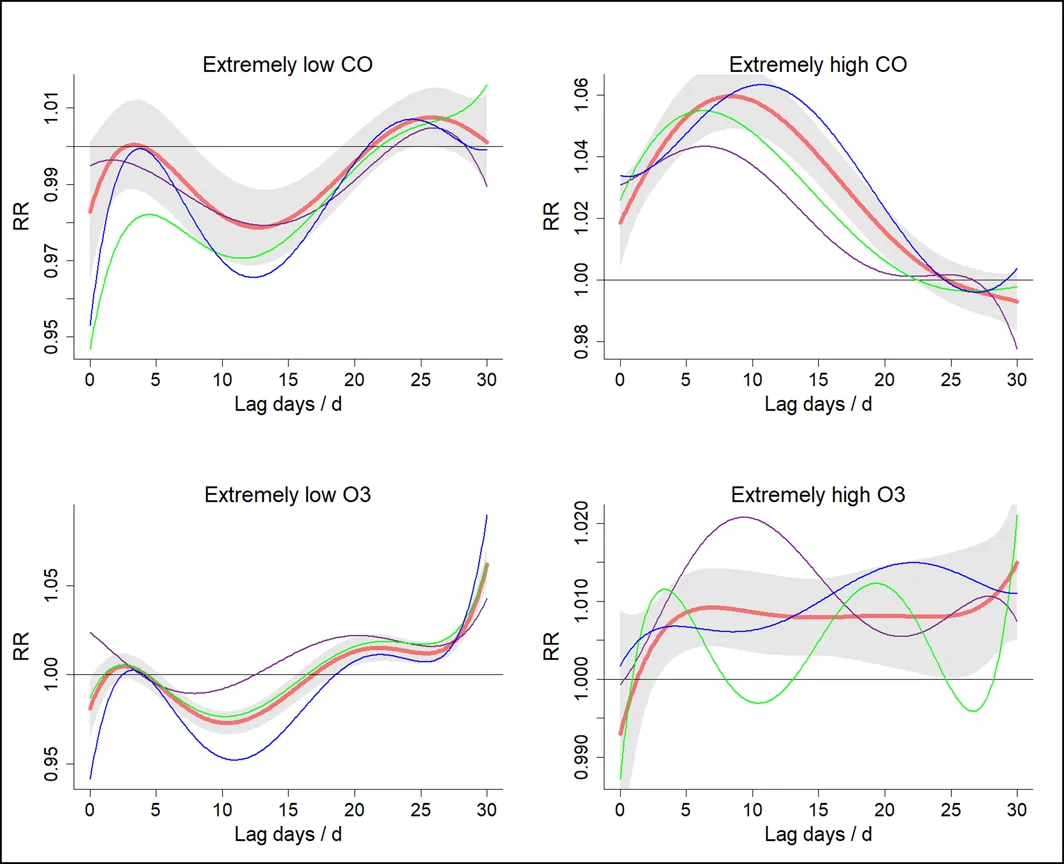
The distributed lag effect of extreme CO and O3 on respiratory diseases at different lag days (Red: The entire population; Purple: The population under 15 years old; Green line: The population aged 15–65 years old; Blue: The population over 65 years old).

Figure 13 indicates that the impacts of CO and $O_{3}$ on respiratory diseases exhibit complex temporal characteristics. Regarding CO, exposure to extremely low concentrations shows a protective effect on all populations within 0–20 days, which may be related to the antioxidant stress response induced by low concentrations of CO. However, exposure to extremely high concentrations of CO leads to an increase in the risk of respiratory diseases and reaches a peak 5–10 days after exposure. This is likely related to the high affinity of CO for hemoglobin, which causes tissue hypoxia and aggravates the risk of respiratory diseases through mechanisms such as inducing oxidative stress and disrupting the barrier function of alveolar epithelial cells (Liu et al., 2018). The impact of $O_{3}$ is more complex. In the initial stage of exposure (5–15 days), low concentrations of $O_{3}$ lead to a trend of reduced disease risk, which may be related to the adaptive cytoprotective mechanisms induced by $O_{3}$. However, as time goes by, this protective effect gradually weakens, and the risk starts to rise continuously after 15 days. Exposure to high concentrations of $O_{3}$ shows a continuous risk accumulation effect, and children under 15 years old are the most sensitive, which may be related to the incomplete development of the respiratory system and relatively weak lung function in children. It is worth noting that there are significant differences in the response patterns of people of different age groups to $O_{3}$, and these differences may be related to factors such as age‐related immune function, metabolic capacity, and exposure behavior patterns (Cortiñas et al., 2025).

**Figure 14:**
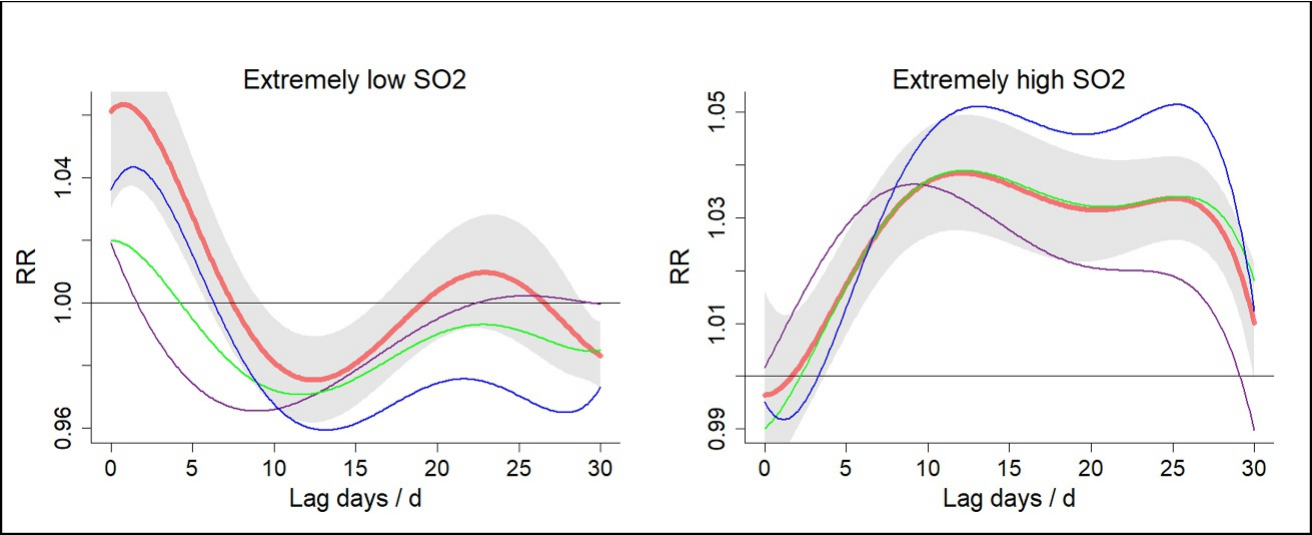
The distributed lag effect of extreme ${\text{SO}}_{2}$ on respiratory diseases at different lag days (Red: Whole population; Purple: Population under 15 years old; Green line: Population aged 15–65 years old; Blue: Population over 65 years old).

The results of Figure 14 show that the impact patterns of extremely low concentrations of ${\text{SO}}_{2}$ on all populations are basically the same: on the first day after exposure, the relative risk of respiratory diseases reaches its maximum value, and then gradually decreases, reaching its lowest point at 10–15 days, and then slowly rebounds (Cao et al., 2022). This trend of “first rising, then falling, and then rebounding” may be related to the acute irritating effect of low concentrations of ${\text{SO}}_{2}$ on the respiratory tract. In the initial stage, it triggers an inflammatory response, and then the body gradually adapts, but long‐term exposure may lead to chronic damage. However, when the concentration of ${\text{SO}}_{2}$ increases, the impact pattern is completely different. After exposure to high concentrations of ${\text{SO}}_{2}$, the relative risk of respiratory diseases slowly rises over time, reaches its peak at about 10 lag days, remains at a high level for about 15 days, and then begins to slowly decline. This persistent high‐risk state may be related to the deep damage of high concentrations of ${\text{SO}}_{2}$ to the respiratory tract, including the destruction of alveolar epithelial cells and the suppression of immune function. Among them, the elderly population over 65 years old is significantly more sensitive to ${\text{SO}}_{2}$ than other age groups. Whether exposed to low or high concentrations of ${\text{SO}}_{2}$, the relative risk of respiratory diseases in the elderly population is significantly increased. This may be related to the degradation of respiratory function, the decline in immunity, and the presence of multiple chronic underlying diseases in the elderly, making their defense ability against air pollutants relatively weak. In addition, the elderly often have the situation of multiple drug use, and the interaction between drugs and pollutants may also exacerbate health risks (Wigenstam et al., 2016).

**Figure 15:**
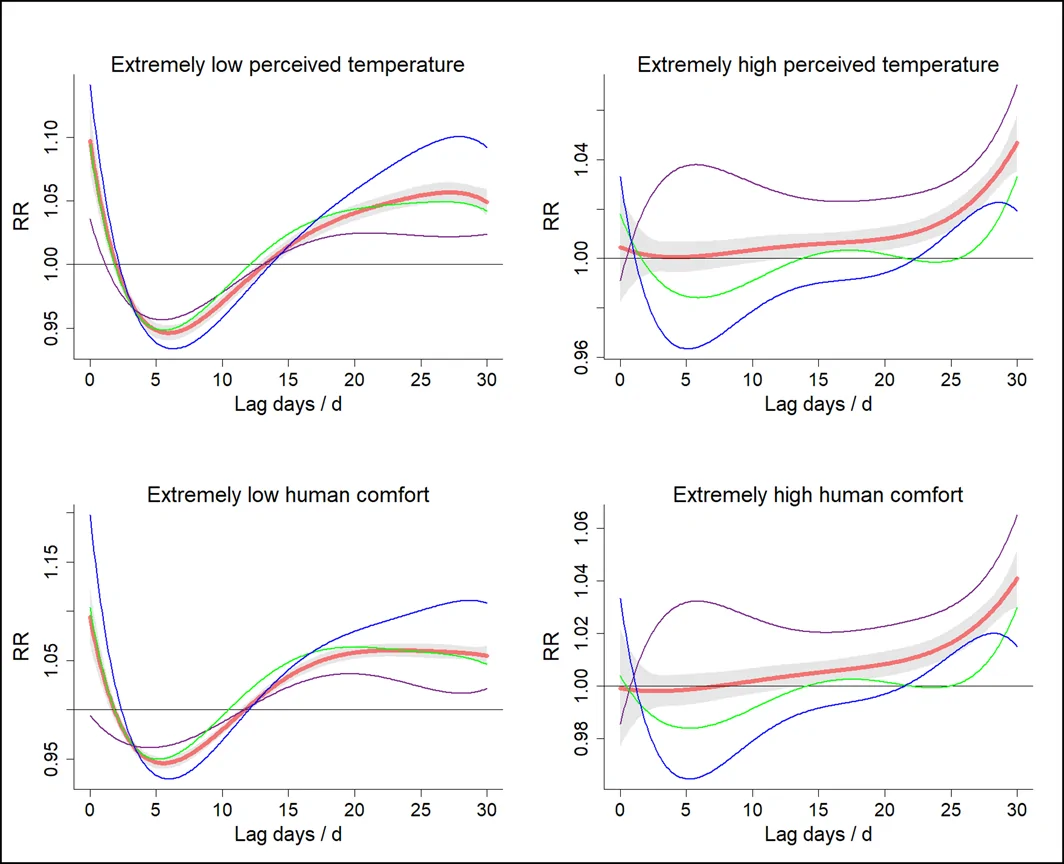
The distributed lag effect of extreme perceived temperature and human comfort on respiratory diseases at different lag days (Red: The entire population; Purple: The population under 15 years old; Green line: The population aged 15–65 years old; Blue: The population over 65 years old).

**Figure 16:**
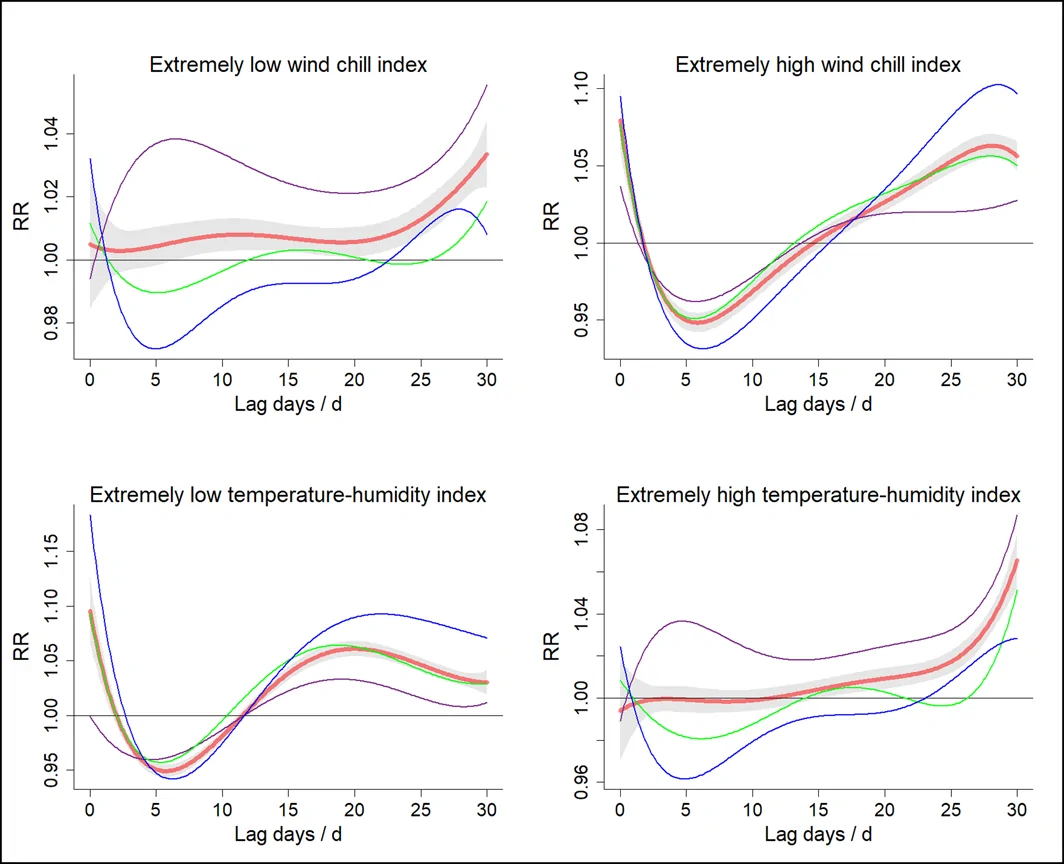
The distributed lag effect of extreme wind chill index and temperature‑humidity index on respiratory diseases at different lag days (Red: The entire population; Purple: The population under 15 years old; Green line: The population aged 15–65 years old; Blue: The population over 65 years old).

The perceived temperature, human comfort, and the temperature‐humidity index, since their calculations are based on the same meteorological indicators, the impact patterns of their extreme values on the relative risk of respiratory diseases are relatively consistent (as shown in Figures 15 and 16). When these indicators are at extremely low levels, the relative risk of respiratory diseases reaches its maximum value on the day of exposure, then drops rapidly, and reaches a trough within 5–10 days, and then slowly rebounds. This trend of “first rising, then falling, and then rebounding” may be related to the acute stimulation of the respiratory tract by extremely low temperature or low humidity environments. In the initial stage, it triggers mucosal dryness and inflammatory reactions, and then the body gradually adapts, but long‐term exposure may lead to chronic damage (D’Amato et al., 2018).

However, when these indicators are at extremely high levels, there are significant differences in the response patterns of people of different age groups. For children under 15 years old, the relative risk of respiratory diseases is the lowest on the day of exposure, then reaches a peak at about 5 lag days, and then slowly decreases, but the risk value rises again at 25 lag days. This delayed effect may be related to the immature development of the immune system in children, resulting in a relatively slow response to extremely high temperature or high humidity environments and being vulnerable to secondary infections (Kim & Lee, 2019). In contrast, the response patterns of people in other age groups under extremely high environmental conditions are more direct: the relative risk of respiratory diseases reaches the highest value on the day of exposure, then decreases rapidly, reaches the lowest point at 5 lag days, and then slowly rebounds. This rapid response may be related to the higher sensitivity of the respiratory tracts of adults and the elderly to extreme environments, especially the elderly who may have underlying respiratory diseases, further exacerbating the acute risk (Rocklöv et al., 2011).

Overall, the extreme values of the perceived temperature, human comfort, temperature‐humidity index, and wind chill index not only exhibit obvious time lag effects on respiratory diseases but also show significant age differences. This finding suggests that under extreme weather conditions, differentiated health protection measures should be taken for people of different age groups. In particular, more attention should be paid to the health risks of children and the elderly.

### 3.4 Analysis of the Spatial Heterogeneity of Influencing Factors of Respiratory Diseases

After the multicollinearity test, variables with strong collinearity were removed. All explanatory variables were standardized (z‐score normalization) before GTWR model establishment to eliminate the influence of different units and magnitude. Therefore, the absolute values of GTWR coefficients can be used to compare the relative importance of each predictor. Temperature, air pressure, relative humidity, ${\text{PM}}_{10}$, CO, $O_{3}$, ${\text{SO}}_{2}$, wind chill index, perceived temperature, and SDI indicators were selected as influencing factors and incorporated into the construction of the GTWR model. The $R^{2}$ of the GTWR reached 66.56%, indicating that it can well explain the influence of various influencing factors on the HRRD in each county and district of Jiangsu Province.

Table 2 presents the statistical results of the fitting coefficients for each influencing factor. According to the findings, the absolute values of the average fitting coefficients are ordered as follows: temperature (−1.94) > perceived temperature (1.42) > SDI (0.55) > wind chill index (−0.32) > CO (−0.20) > ${\text{PM}}_{10}$ (0.13) > ${\text{SO}}_{2}\;\left(-0.13\right)$ > air pressure (−0.12) > $O_{3}\;\left(-0.04\right)$ > relative humidity (−0.02). These results indicate that temperature has the greatest impact on the HRRD, exhibiting a negative correlation—a finding consistent with previous results in this study.

**Table 2:** Data Statistics of Fitting Coefficients of Influencing Factors of Respiratory Diseases in Jiangsu Province

Variable	Mean	Std	Min	25%	50%	75%	Max
CO	−0.20	0.34	−1.43	−0.32	−0.15	0.05	0.38
$O_{3}$	−0.04	0.16	−0.35	−0.14	−0.07	0.00	0.70
${PM}_{10}$	0.13	0.15	−0.13	0.02	0.11	0.22	0.72
${SO}_{2}$	−0.13	0.28	−1.18	−0.25	−0.11	0.02	0.81
Pressure	−0.12	0.45	−1.82	−0.11	−0.03	0.05	0.63
Temperature	−1.94	2.51	−12.61	−3.08	−0.97	−0.28	1.82
RH	−0.02	0.15	−0.36	−0.14	−0.02	0.09	0.31
ET	1.42	3.12	−4.63	−0.71	0.48	3.40	10.15
THI	−0.32	2.30	−5.87	−1.65	−0.29	1.31	5.80
SDI	0.55	0.51	−0.05	0.24	0.41	0.62	2.59

**Figure 17:**
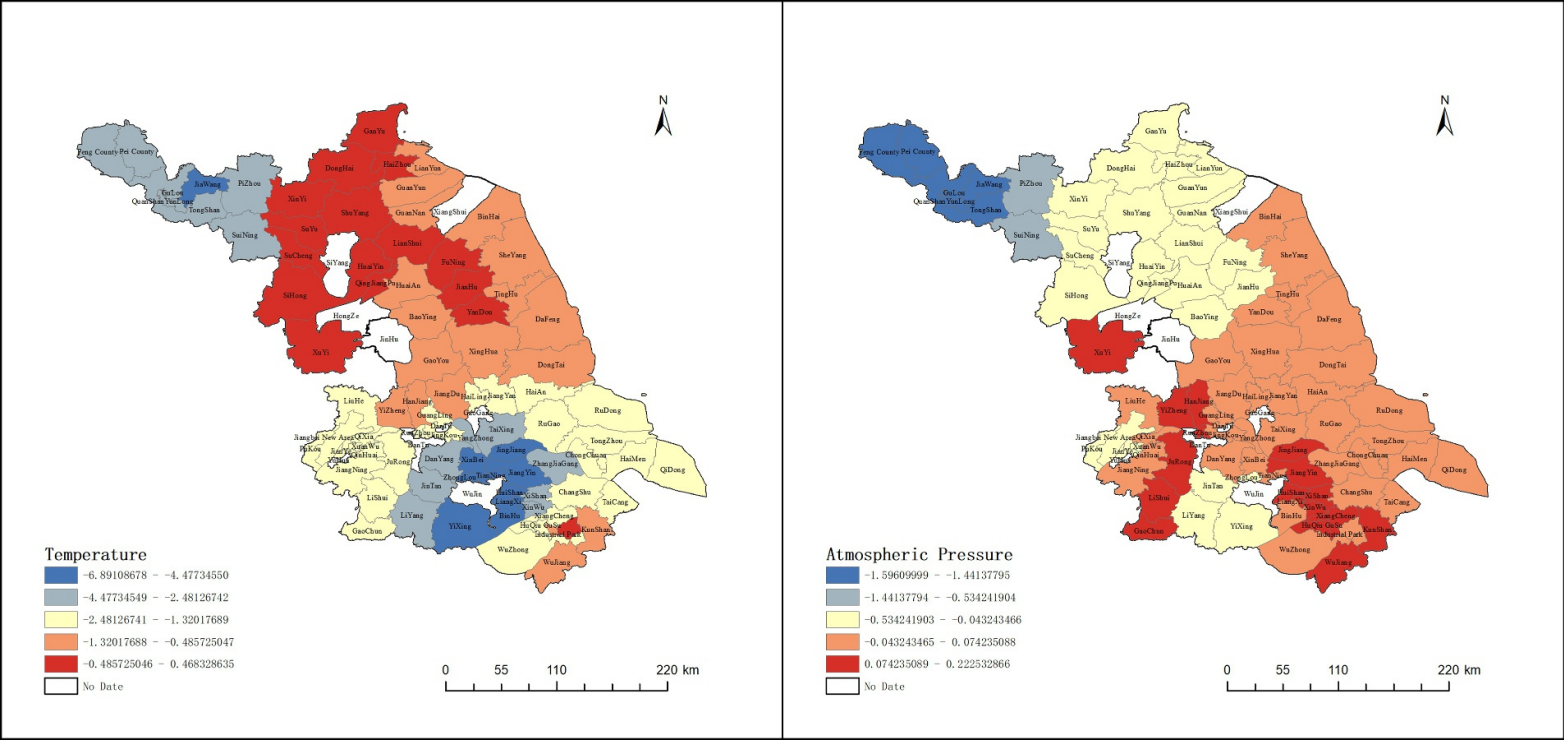
The spatial distribution of the fitting coefficients of the impacts of air temperature and air pressure on respiratory diseases.

As can be seen from Figure 17, the impacts of temperature and air pressure on the HRRD show significant spatial heterogeneity. In terms of spatial distribution, the HRRD in most areas of Jiangsu Province is negatively correlated with temperature, that is, the lower the temperature, the higher the hospitalization rate. This negative correlation effect shows obvious agglomeration characteristics in space: counties and districts that are close to each other geographically tend to show similar temperature ‐ influence patterns. For example, the areas with the highest negative correlation are mainly concentrated in Tianning District, Jiangyin City, and Huishan District in the south, as well as Jiawang District and Tongshan District in the north. In contrast, the areas where temperature has less impact on the HRRD are mainly concentrated in the northern and central parts of Jiangsu Province, and also include the Industrial Park and Kunshan City in the southern part. In some central and southern parts of Jiangsu Province (the red areas in the figure), there is a positive correlation between air pressure and the HRRD, that is, the higher the air pressure, the higher the hospitalization rate. However, in Feng County, Pei County and other places in the northern region, there is a negative correlation between air pressure and the HRRD. Instead, the increase in air pressure is associated with the decrease in the hospitalization rate. Province on meteorological factors.

**Figure 18:**
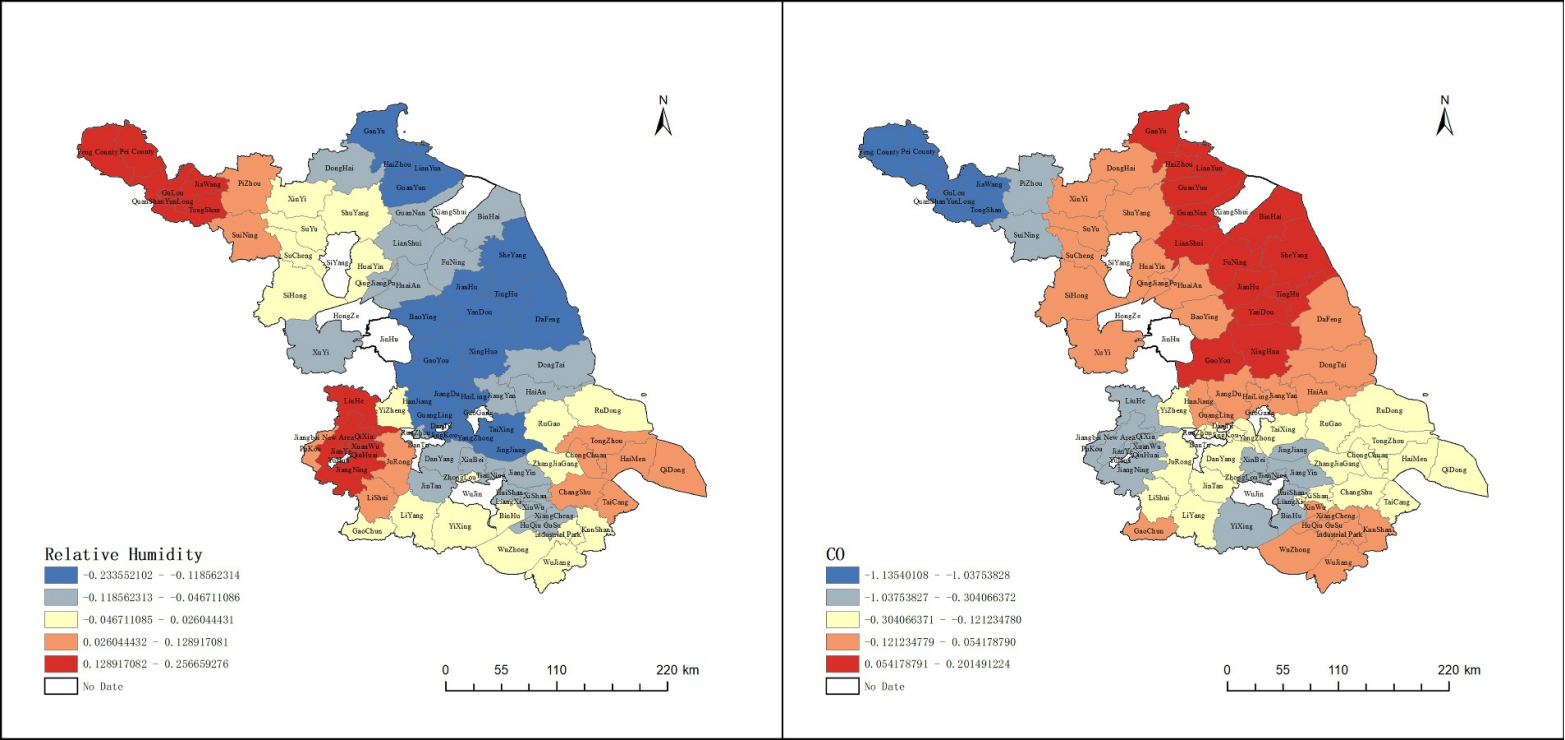
The spatial distribution of the fitting coefficients of the impacts of relative humidity and CO on respiratory diseases.

The results of Figure 18 demonstrate that the relationship between respiratory diseases and relative humidity exhibits distinct spatial heterogeneity within Jiangsu Province, presenting a distribution pattern of positive correlation in the north and south and negative correlation in the central area. Specifically, the counties and districts in Xuzhou and Nanjing show a high positive correlation with relative humidity, meaning that the higher the relative humidity, the higher the incidence rate of respiratory diseases. In contrast, the central region shows a negative correlation, where an increase in relative humidity is instead associated with a decrease in the disease incidence rate. In summer and winter, a high‐humidity environment is likely to exacerbate the inflammatory response of the respiratory mucosa and increase the risk of pathogen transmission, thereby leading to an increase in the incidence rate of respiratory diseases (Luo et al., 2020). Additionally, in northern regions such as Xuzhou, during the winter heating period, there is a large temperature difference between indoors and outdoors. In a high‐humidity environment, on one hand, it promotes the hygroscopic growth of pollutants like ${\text{PM}}_{2.5}$, increasing the deposition efficiency of inhalable particles (Chen et al., 2021). On the other hand, it prolongs the survival time of pathogens such as influenza viruses in aerosols (Irwin et al., 2011). Southern regions like Nanjing, due to their unique “furnace‐like” climate characteristics, the high‐temperature and high‐humidity environment in summer easily induces airway hyperresponsiveness (Wang et al., 2022). Coupled with the superimposed effects of the urban heat island effect and high population density, it significantly amplifies the negative effects of humidity on respiratory health (H. Huang et al., 2019). In contrast, the central region has relatively dry climate conditions and low humidity. The lower humidity may reduce the survival time of pathogens in the air and lower the risk of respiratory tract infections (Mäkinen et al., 2009). Regarding the impact of CO, in the central region, especially in coastal counties and districts such as Lianyungang and Yancheng, there is mostly a positive correlation between the CO concentration and the influencing factors, which may be related to the concentration of industrial emission sources and relatively limited diffusion conditions in this area. It is worth noting that counties and districts in the northern region, such as Feng County and Pei County, show significant negative correlation characteristics, presumably due to the higher vegetation coverage in this region, which results in a stronger absorption of CO. In contrast, in the southern region, except for the weak correlation in the counties and districts of Suzhou, the rest of the areas generally exhibit a strong negative correlation.

**Figure 19:**
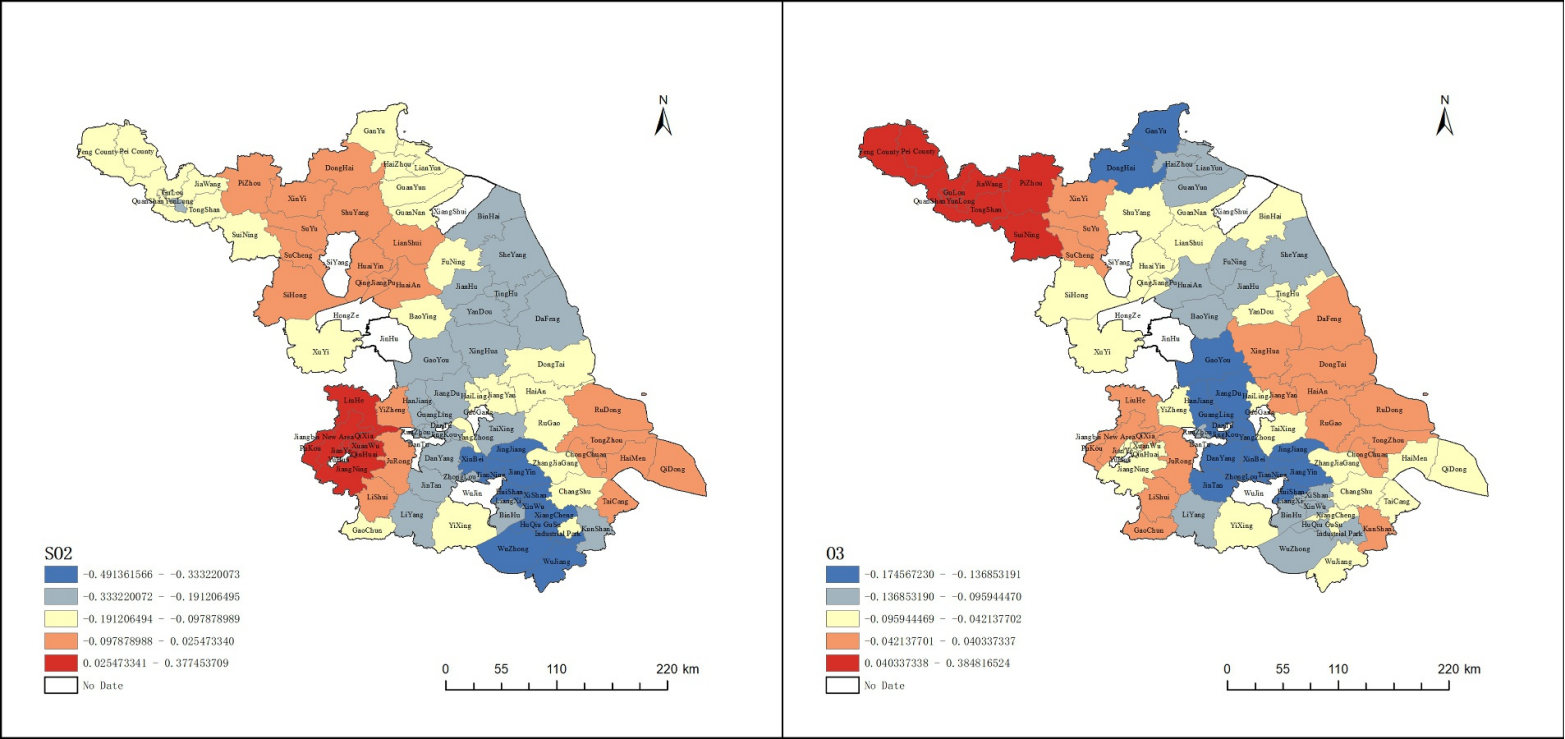
The spatial distribution of the fitting coefficients of the impacts of ${\text{SO}}_{2}$ and $O_{3}$ on respiratory diseases.

From the overall results of Figure 19, most regions show a negative correlation with ${\text{SO}}_{2}$, among which the negative correlation in southern Jiangsu regions such as Suzhou and Wuxi is the most prominent. In contrast, the Nanjing region presents a unique positive correlation, which may be related to its status as a regional central city, the continuously high intensity of industrial activities, and the rapid increase in the number of motor vehicles. In addition, the spatial distribution of the fitting coefficients of the impact of ${\text{SO}}_{2}$ does not show obvious regular characteristics. This discrete distribution pattern may reflect the comprehensive influence of the regional economic development level, the characteristics of the energy structure, and the intensity of pollution control (Feng et al., 2020). The impact of $O_{3}$ is more complex. Except for the positive correlation in the Xuzhou region, most other regions show a negative correlation, and there is no obvious geographical distribution pattern.

**Figure 20:**
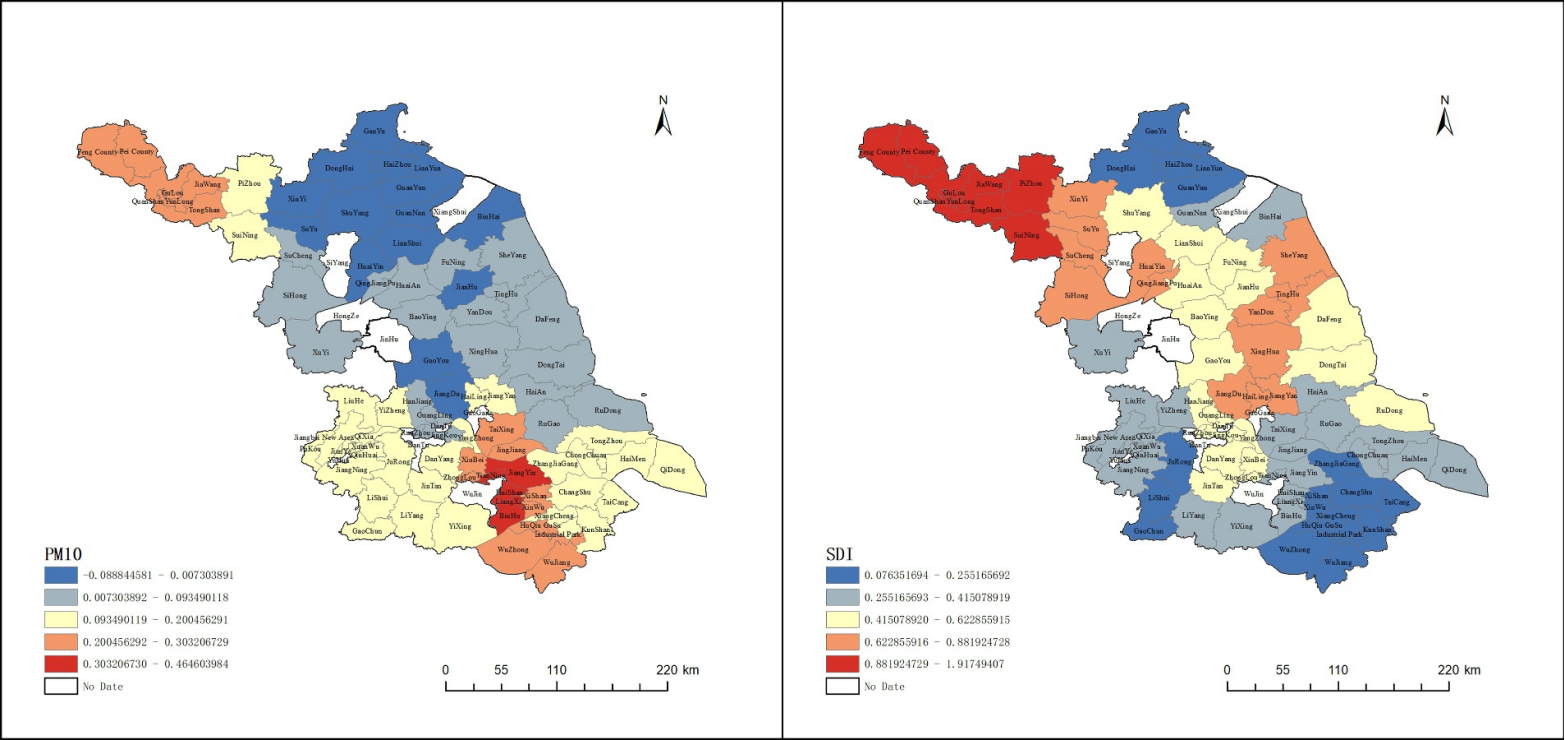
The spatial distribution of the fitting coefficients of the impacts of ${\text{PM}}_{10}$ and SDI on respiratory diseases.

Figure 20 shows that ${\text{PM}}_{10}$ has a positive correlation with respiratory diseases in most areas of Jiangsu Province, and the correlation in the southern region is significantly stronger than that in the northern region. This may be related to the higher urbanization level, larger population density, and greater intensity of industrial activities in the southern region. The areas with the highest positive correlation are concentrated in some counties and districts of Wuxi City. This phenomenon may be related to the special geographical environment and industrial structure of this area: located in the Taihu Lake Basin, it is prone to the accumulation of pollutants due to the influence of local circulation (Zhan et al., 2025). There is a significant positive correlation between the SDI and the HRRD in each county and district of Jiangsu Province, and it shows obvious spatial gradient characteristics. Specifically, the influence coefficient of the SDI gradually increases from south to north. This may be due to the relatively insufficient medical resource allocation and lower accessibility to medical services in northern Jiangsu, which amplifies the impact of the social deprivation index on health.

**Figure 21:**
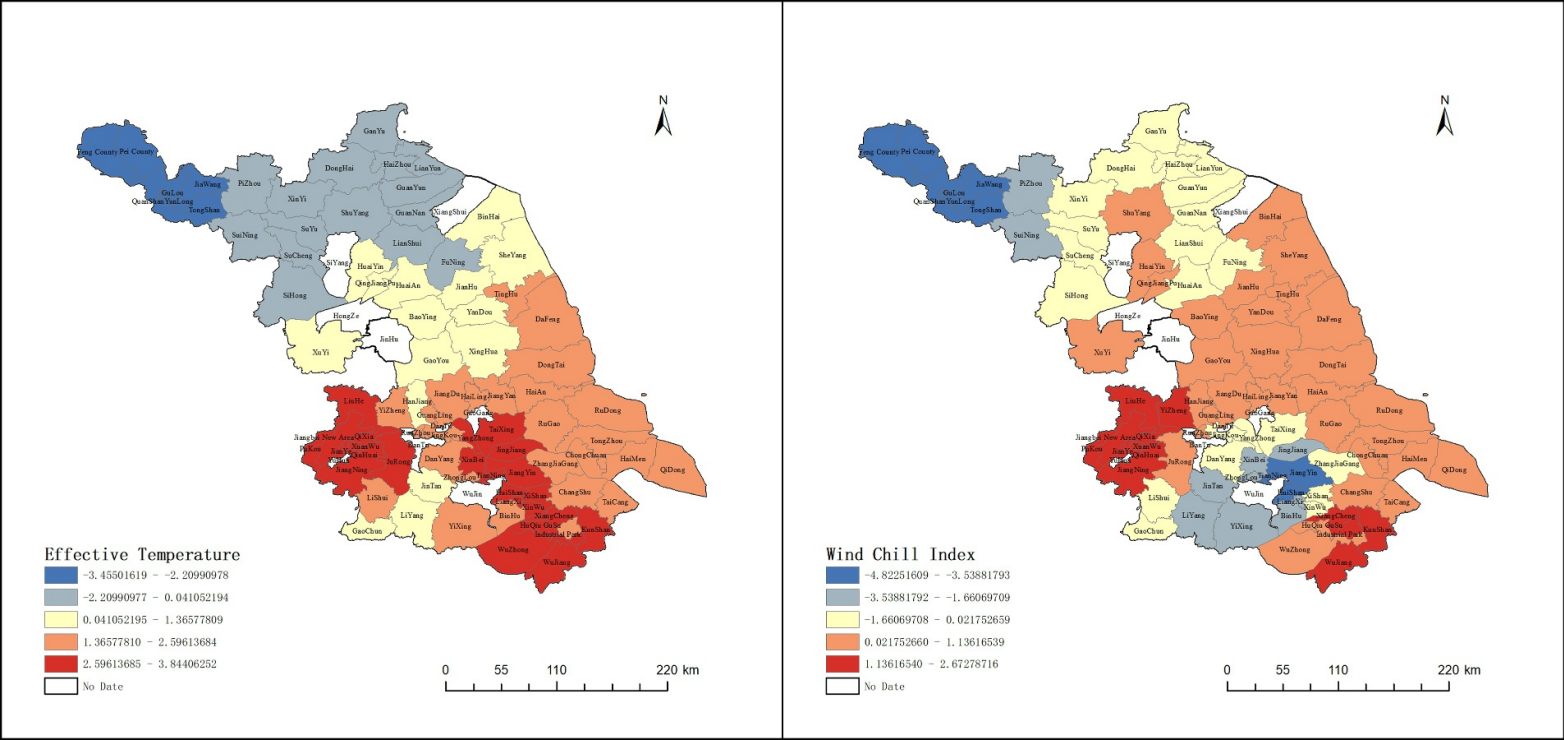
The spatial distribution of the fitting coefficients of the impacts of the perceived temperature and the wind chill index on respiratory diseases.

As can be seen from Figure 21, the impact of the perceived temperature on the HRRD shows significant spatial heterogeneity. In terms of the spatial distribution pattern, the northern region mainly shows a negative correlation, while the southern region shows a positive correlation, and there is an obvious north‐south gradient change. The cold and dry climate characteristics in the northern region make lower temperatures more likely to induce respiratory diseases, while the high‐temperature and high‐humidity environment in the southern region may increase the burden on the respiratory system (Li et al., 2019). In terms of the overall distribution characteristics, the spatial impact pattern of the wind chill index is somewhat similar to that of the perceived temperature, both showing obvious north‐south differences. The northern region has a negative correlation with the wind chill index, and the southern region has a positive correlation. This spatial distribution characteristic may be mainly related to the regional climate background and the adaptability of residents. The cold and windy winter in the northern region makes a lower wind chill index more likely to induce respiratory diseases. In contrast, the relatively mild winter climate in the southern region makes the health impact of the wind chill index relatively weak. However, some counties and districts in Wuxi and Changzhou are contrary to the surrounding areas, with a highly negative correlation between the wind chill index and respiratory diseases. The reason may be that the high population density and urbanization level may form a unique local microclimate, weakening the actual impact of the wind chill index (X. Huang & Zhang, 2025).

## 4 Conclusion

This study employed DLNM and GTWR to systematically analyze the spatio‐temporal heterogeneous impacts of meteorological factors (extreme temperature, humidity, air pressure), air pollutants (${\text{PM}}_{2.5}$, ${\text{PM}}_{10}$, CO, $O_{3}$, ${\text{SO}}_{2}$), and comprehensive climate indicators on respiratory diseases. Temporally, DLNM quantified distinct lag effects across age groups: for those under 15, extremely high temperature's relative risk (RR) peaks around 5 lag days, while for people aged 15–65, extreme low temperature shows a “U‐shaped” RR change (peaking at 0–2 lag days, RR = 1.10). High concentrations of $O_{3}$ cause continuous risk accumulation (most severe for <15s, RR = 1.20), and high ${\text{SO}}_{2}$ concentrations have an RR peak at 10 lag days (RR = 1.25), with over‐65s most sensitive; notably, extremely high humidity often has a protective effect (RR < 1) within 30 lag days. Spatially, GTWR revealed marked differences in Jiangsu Province: temperature has a strong negative correlation with hospitalization rates in southern regions (e.g., Tianning District) but weak impact in the north; ${\text{PM}}_{10}$ shows a stronger positive correlation with hospitalization rates in the south (especially Wuxi); SDI (Socio‐Demographic Index) has a positive correlation that strengthens from south to north; air pressure, humidity, and CO exhibit complex spatial patterns (e.g., humidity correlates positively with hospitalization rates in Xuzhou/Nanjing but negatively in central Jiangsu). This research provides a critical scientific basis for formulating targeted, regional prevention and control strategies—for example, prioritizing elderly protection in areas with high ${\text{PM}}_{2.5}$ risks and adjusting interventions based on southern Jiangsu's temperature‐sensitive hospitalization trends. These findings also facilitate the establishment of regional early warning mechanisms, optimize healthcare resource allocation, and guide targeted public health interventions for susceptible groups, delivering practical implications for local public health governance. Limitations include Jiangsu‐only focus (restricting generalizability) and insufficient analysis of multi‐factor interactions. Future research will expand to more regions and longer time series, explore synergistic effects of multiple factors, and integrate more socioeconomic factors (e.g., education level, healthcare accessibility) through methods like cross‐sector data integration and multi‐source information fusion to deepen understanding of disease drivers and optimize public health interventions.

## Conflict of Interest

The authors declare no conflicts of interest relevant to this study.

## Data Availability

The meteorological data for the same period were collected from the European Centre for Medium‐Range Weather Forecasts (ECMWF) fifth‐generation global atmospheric reanalysis (ERA5) (Hersbach et al., 2023). Air pollutant data were obtained from the China High‐Resolution High‐Quality Near‐Surface Air Pollutants Data set, released by the National Tibetan Plateau Data Center (Wei Jing, 2024).Respiratory disease inpatient statistics were obtained from partial hospitals in 89 counties and districts of Jiangsu Province. Licensed only for this research, the data cannot be publicly shared due to access limitations.
